# Clinical and cost-effectiveness of Knee Arthroplasty versus Joint Distraction for Osteoarthritis (KARDS): protocol for a multicentre, phase III, randomised control trial

**DOI:** 10.1136/bmjopen-2022-062721

**Published:** 2022-06-30

**Authors:** Cerys Joyce Tassinari, Ruchi Higham, Isabelle Louise Smith, Susanne Arnold, Ruben Mujica-Mota, Andrew Metcalfe, Hamish Simpson, David Murray, Dennis G McGonagle, Hemant Sharma, Thomas William Hamilton, David R Ellard, Catherine Fernandez, Catherine Reynolds, Paul Harwood, Julie Croft, Deborah D Stocken, Hemant Pandit

**Affiliations:** 1 Clinical Trials Research Unit, Leeds Institute of Clinical Trials, University of Leeds, Leeds, UK; 2 Warwick Clinical Trials Unit, University of Warwick Warwick Medical School, Coventry, UK; 3 Leeds Institute of Health Sciences, University of Leeds, Leeds, UK; 4 Trauma and Orthopaedics, University Hospitals Coventry and Warwickshire NHS Trust, Coventry, UK; 5 Department of Orthopaedics and Trauma, University of Edinburgh, Edinburgh, UK; 6 Nuffield Department of Orthopaedics Rheumatology and Musculoskeletal Sciences, University of Oxford, Oxford, Oxfordshire, UK; 7 Chapel Allerton Hospital, Leeds Institute of Rheumatic and Musculoskeletal Medicine, University of Leeds, NIHR Leeds Biomedical Research Centre, Leeds, UK; 8 Department of Orthopaedics, Hull and East Yorkshire Hospitals NHS Trust, Hull, Kingston upon Hull, UK; 9 University of Leeds, Leeds Institute of Medical Research, Leeds, West Yorkshire, UK; 10 Chapel Allerton Hospital, Leeds, West Yorkshire, UK

**Keywords:** knee, musculoskeletal disorders, orthopaedic & trauma surgery, statistics & research methods

## Abstract

**Introduction:**

Knee replacement (KR) is a clinically proven procedure typically offered to patients with severe knee osteoarthritis (OA) to relieve pain and improve quality of life. However, artificial joints fail over time, requiring revision associated with higher mortality and inferior outcomes. With more young people presenting with knee OA and increasing life expectancy, there is an unmet need to postpone time to first KR. Knee joint distraction (KJD), the practice of using external fixators to open up knee joint space, is proposed as potentially effective to preserve the joint following initial studies in the Netherlands, however, has not been researched within an NHS setting. The KARDS trial will investigate whether KJD is non-inferior to KR in terms of patient-reported postoperative pain 12 months post-surgery.

**Methods and analysis:**

KARDS is a phase III, multicentre, pragmatic, open-label, individually randomised controlled non-inferiority trial comparing KJD with KR in patients with severe knee OA, employing a hybrid-expertise design, with internal pilot phase and process evaluation. 344 participants will be randomised (1:1) to KJD or KR. The primary outcome measure is the Knee Injury and Osteoarthritis Outcomes Score (KOOS) pain domain score at 12 months post-operation. Secondary outcome measures include patient-reported overall KOOS, Pain Visual Analogue Scale and Oxford Knee Scores, knee function assessments, joint space width, complications and further interventions over 24 months post-operation. Per patient cost difference between KR and KJD and cost per quality-adjusted life year (QALY) gained over 24 months will be estimated within trial, and incremental cost per QALY gained over 20 years by KJD relative to KR predicted using decision analytic modelling.

**Ethics and dissemination:**

Ethics approval was obtained from the Research Ethics Committee (REC) and Health Research Authority (HRA). Trial results will be disseminated at clinical conferences, through relevant patient groups and published in peer-reviewed journals.

**Trial registration number:**

ISRCTN14879004; recruitment opened April 2021.

**Strengths and limitations of this study**

- The Knee Arthroplasty versus Joint Distraction for Osteoarthritis trial is a pragmatic trial design using standardised surgical and assessment techniques, robust reporting and safety mechanisms and appropriate sample size.
- A hybrid-expertise-based design has been adopted to ensure feasibility of the trial while accounting for surgeon experience and potential lack of individual equipoise.
- A surgical manual will document each trial procedure highlighting mandatory components according to recommended guidance for surgery trials. This will allow comprehensive reporting of the interventions delivered during the trial.
- Due to the nature of the interventions, the trial personnel and participants are not blinded to treatment allocation.

## Background

Osteoarthritis (OA) is the most common musculoskeletal condition that affects joints, causing pain, joint dysfunction and significant quality of life (QoL) impact. With rising obesity rates and ageing population, the number of people presenting with knee OA is increasing.^1^

Patients with severe knee OA experiencing joint symptoms that substantially impact QoL are typically offered knee replacement (KR) to relieve pain and improve mobility. KR is clinically proven and cost-effective;^2^ however, artificial joints have a finite life span. If KR fails, revision is complex, costly and associated with higher morbidity, mortality and inferior outcomes.^3–5^

The James Lind Alliance established that defining the optimum timing of joint replacement, in order to achieve the best outcome is a significant patient concern.^6^ The number of young patients (55 years or less) undergoing KR are increasing,^5^ and risk of failure is disproportionately higher in the young and active. A combined endpoint analysis including revision, poor function and significant pain has shown KR success to be as low as 59% after 12 years in patients 60 years or less.^7^ Increasing life expectancy and the growing number of younger patients means there is a need for treatment, which postpones the time to first KR, without compromising QoL or hampering ability to undergo KR at a later stage.^7 8^ As in joint replacement in general, it is unknown whether treatment options preserving the joint are cost-effective.^9 10^

Knee joint distraction (KJD), the practice of placing an external fixator across a synovial joint and pulling the joint surfaces apart approximately 5 mm for ~6 weeks, has been proposed as a potentially effective alternative to preserve the joint. The aim is to harness intrinsic joint-repair potential, providing cartilage repair and normalisation of subchondral bone abnormalities.^11^ KJD is not currently widely used in the UK, and no trials have been conducted in the NHS. Initial studies conducted in the Netherlands suggest it is a safe and potentially effective treatment.^12–14^ One small trial suggested KJD to be non-inferior to total KR in function^15–17^ and another predicted that it could save over 30% of revision KRs.^18^ With a willingness to pay €20 000 per quality-adjusted life year (QALY), KJD was shown to be cost-effective in over 75% cases for all age groups and over 90% in the young (55 years or less).^19^

### Rationale

There is strong scientific basis for KJD with excellent cartilage regeneration in experimental OA with joint offloading procedures.^20^ Given the preliminary clinical data and underpinning science, KJD could be an alternative therapy to KR for younger patients, but current evidence is limited. Patient feedback highlighted the key priority for those in this age group is retaining their own knee, at expense of some residual knee pain. If KJD is shown to be safe, non-inferior to KR in terms of pain and cost-effective in the NHS, then it could be routinely offered to patients 65 years or less, delaying need for KR and potentially avoiding revision surgery. This is the aim of the Knee Arthroplasty versus joint distraction for osteoarthritis (KARDS) trial.

## Methods and design

### Objectives

The primary objective is to conduct a multicentre trial to investigate clinical effectiveness of KJD compared with KR in patients aged 65 years or less, with symptomatic knee OA severe enough to warrant KR, based on patient reported pain 12 months after surgery.

Secondary objectives are to investigate: (1) patient-reported outcomes, (2) clinical outcomes of knee function, (3) complications and need for further intervention, (4) cost-effectiveness, (5) participant experiences, intervention fidelity and barriers to wider implementation.

### Trial design

This publication describes KARDS protocol V.2.0, dated 29th September 2020.

KARDS is a phase III IDEAL stage 3 assessment,^21^ multicentre, pragmatic, open-label, 1:1, two-arm individually randomised controlled trial, with embedded 12-month internal pilot phase.

The internal pilot phase will incorporate a qualitative process evaluation to identify potential barriers to recruitment and any challenges experienced in maintaining intervention fidelity. As part of the process evaluation, qualitative semi-structured interviews will be undertaken with clinicians, trial staff and participants to explore experiences of trial involvement and intervention acceptability during the pilot phase and throughout the main trial. Progression at the end of the pilot phase will be based on (1) recruitment and dropout rates, (2) safety, (3) the process evaluation.

#### Trial setting and recruitment

Participants will be recruited from secondary care orthopaedic centres following general practitioner (GP) or specialist referral. Potentially eligible participants will be identified by the attending clinical team from orthopaedic outpatient clinics and theatre lists. Following information provision, patients will be given the opportunity to discuss the trial with their family, friends and healthcare professionals before being invited to participate.

Informed consent will be obtained by the Principal Investigator (PI) or appropriate, delegated, healthcare professional as detailed on the Authorised Personnel Log, in accordance with the principles of Good Clinical Practice and Declaration of Helsinki 1996.

All sites must be able to deliver both KR and KJD. As KJD is not a standard technique used in knee surgery, not all surgeons will have the required experience, and some surgeons may not be in individual equipoise despite there being centre equipoise. A hybrid expertise-based design, where surgeons are categorised into ‘delivery units’ based on experience, addresses both issues.

There are two delivery unit categories based on the interventions surgeons are authorised to perform within the trial: (1) *single delivery units* consist of surgeons authorised to deliver KJD **or** KR, where the surgeon performing the procedure will be chosen after randomisation, depending on the allocation or (2) *dual delivery unit*s, consisting surgeons authorised to deliver KJD **and** KR where a randomised participant may receive either operation by the same surgeon.

#### Eligibility

Surgeon eligibility: participating surgeons must either be a consultant orthopaedic surgeon or perform the procedure under direct consultant supervision. To deliver KR within KARDS, a surgeon must have performed ≥10 KRs in the past 12 months as the primary surgeon. To deliver KJD within KARDS, they must have performed ≥10 external fixations during their career as the primary surgeon or completed a limb reconstruction fellowship.

Patient eligibility: criteria are minimised to ensure inclusivity and generalisability. Adult patients are eligible if aged ≤65 years requiring KR and meet the criteria in table 1.

**Table 1:** Patienteligibility criteria

Inclusion criteria	Exclusion criteria
Age≥18 years and ≤65 years at time of signing the Informed Consent form	Bone density not sufficient to support pins for 6 weeks*
Symptoms (pain and/or reduced function) severe enough to warrant knee replacement*	Isolated patella-femoral OA*
Pre-operative leg alignment not requiring correction*	Complete joint space obliteration in both medial and lateral tibio-femoral compartments as seen on weight bearing AP knee radiograph
Intact collateral knee ligaments*	A known diagnosis of inflammatory arthritis
Fixed flexion deformity ≤10^o^	Presence of a previous joint replacement in any limb
	Surgical treatment of involved knee within the past 6 months (excluding arthroscopy)
	Previous knee joint distraction on the involved knee
	Previously participated in the KARDS trial
	Weight >120 kg
	Pregnant or lactating (confirmed by participant)
	Active cancer (currently diagnosed and under treatment)
	Unable to complete all trial procedures (eg, attend follow-up visits, complete questionnaires)
	Unable to provide informed consent (cognitive disorder such as dementia, psychiatric illness)

*In the opinion of the treating clinician.

#### Interventions

A surgical manual will document each trial procedure highlighting mandatory components according to recommended guidance.^22^

##### Intervention (KJD)

A definitive external fixator construct will be used, which allows for controlled linear distraction across the knee joint of 5 mm. The exact nature of the construct will depend on equipment availability at site and surgeon preference. Devices will be approved for trial use by the Trial Management Group.

During surgery, the external fixation frame will be assembled according to frame construct procedures detailed in the surgical manual, with focus on meticulous pin insertion to minimise complication risk. Pins will be placed under fluoroscopic control. Once assembly completes, ≥2 mm and ≤5 mm axial distraction will be applied across the knee joint. A further 1 mm distraction may be applied per day until 5 mm distraction at the joint is confirmed radiographically, or up to 7 days.

External fixators will be removed under general or regional anaesthesia after 6 weeks. Local protocol for pin-site care will be followed and will be documented. Gentle manipulation under anaesthesia to achieve ≥90° of motion will be attempted at the time of fixator removal.

##### Control (KR)

KR surgery will be performed in line with local practice and the surgical manual and will vary depending on implant type and surgeon preference. Surgeons performing the procedure are expected to comply with specific surgical steps for the implant being used as detailed in the manufacturer instructions for use document.

##### Concomitant care and interventions

Pre-operative preparation and post-operative care will be provided to all trial participants in line with the site’s usual protocol for KRs. Decisions about concomitant medications/treatments for symptomatic knee osteoarthritis will be according to local medical plan and clinical management. Details of analgesia and other medication prescribed will be collected throughout trial. Participants may require further intervention for symptomatic knee OA as per routine practice. Further clinical intervention is permitted for all participants and recorded for the trial.

##### Patient and public involvement

KARDS patient and public involvement (PPI) group provided feedback on choice of primary outcome, minimally important difference used in sample size calculations and the decision to not blind participants. PPI representatives on the Trial Management Group provided feedback on the schedule of events for participants.

#### Randomisation and blinding

Participants will be randomised into the trial by an authorised member of site staff, on a 1:1 basis between KJD and KR, based on a minimisation algorithm with random component balanced for delivery unit and OA severity (Kellgren-Lawrence grades 2–3 versus grade 4).^23^ Randomisation will be performed centrally using Leeds Clinical Trials Research Unit (CTRU) automated secure 24-hour randomisation web or telephone service, occurring on the same day as baseline visit, within 6 weeks of the planned surgery date. Clinical assessments and baseline questionnaires will be completed before randomisation with trial specific assessments performed afterwards. Treatment allocation will not be blinded to participants, medical staff or clinical trial staff.

#### Data collection

Clinical data will be collected at baseline, day of surgery, prior to discharge, week 6 (KJD only), and months 3, 12 and 24 post-surgery. Participant completed data will be collected at baseline, day of surgery and months 3, 6, 12 and 24 post-surgery. Full assessment schedule based on the Standard Protocol Items: Recommendations for Interventional Trials (SPIRIT) guidance is^24^ provided in online supplemental material 1.

10.1136/bmjopen-2022-062721.supp1 Supplementary data

Participating sites will maintain a file of essential trial documentation including copies of all completed Case Report Forms (CRFs). Sites will post paper CRFs and electronically transfer trial X-rays to Leeds CTRU. Trial data will be entered onto an electronic database, except post-surgery questionnaires completed using electronic remote data capture by participants or via postal questionnaire.

Data will be monitored for quality and completeness by CTRU. Missing data will be requested from sites until received, confirmed as unavailable or trial analysis begins. The sponsor reserves the right to conduct periodic source data verification to monitor trial integrity.

Participant qualitative interviews will be conducted by telephone, and staff interviews conducted in person or telephone/video conference. Interviews will be audio recorded on an encrypted recorder, anonymised and transcribed verbatim for analysis.

All information collected during the trial will be kept strictly confidential. Information will be held securely on paper and electronically at Leeds CTRU, with process evaluation data held securely on Warwick Clinical Trials Unit (CTU) server. Both will comply with all aspects of the Data Protection Act 2018. If a participant withdraws consent from further trial treatment and/or further data collection, data to the point of withdrawal will remain on file and included in the analysis.

#### Outcome measures

##### Primary outcome measure

The primary outcome measure is Knee Injury and Osteoarthritis Outcomes Score (KOOS) pain score 12 months post-surgery. Pain was indicated by the PPI group as being the most important outcome to them. KOOS is a patient-administered questionnaire, validated for use in patients with knee OA or knee injury,^25^ recorded on a Likert Scale 0–4, transformed to 0 (worst) to 100 (best) scale.

##### Secondary outcome measures

1. *Patient report outcome measures (PROMs) and QoL within 24 months post-surgery*
  - KOOS (overall and at component level).
  - Pain Visual Analogue Scale (VAS).^26 27^
  - Oxford Knee Score (OKS). 28–30
2. *Objective assessment of knee function*
  - Active range of movement.
  - Timed-up-and-go test.^31 32^
3. *Incidence of complications, including infection*
  - Intra-operative complications.
  - Post-operative complications.^33^
4. *Further interventions within 24 months postsurgery*
  - Further surgical interventions including conversion to KR or revision surgery.
5. *KJD’s potential as cartilage regenerative therapy*
  - Joint space width (assessed using standardised fixed flexion PA at 20^o^ X-rays^34^).
6. *Estimate of short-term and long-term cost-effectiveness*
  - EuroQol 5 Dimensions (EQ-5D)-3L questionnaire at 24 months.
  - Health Resource Utilisation and Private Costs questionnaire at 24 months.
  - Incremental costs per QALY gained at 20 years.
7. Implementation processes and intervention fidelity
  - Quantitative (surgical CRF and central review of post-operative X-rays).
  - Qualitative evaluation with surgical and clinical staff.
8. Qualitative evaluation of participant experiences.

### Statistical considerations and analyses

#### Sample size

Power calculations are based on a non-inferiority hypothesis for the primary outcome measure, KOOS pain score. 344 participants (172 per arm) will have 90% power to demonstrate non-inferiority based on an 8 point non-inferiority margin, assuming an SD of 21 points,^2 35–37^ one-sided 2.5% significance level and 15% dropout rate. The non-inferiority margin was agreed by clinical and patient co-applicants based on being 33% less than the 12 point minimally important difference observed in previous trials,^18 38–40^ clinical co-applicant experience and PPI focus group feedback. No adjustment has been made to accommodate surgeon learning curve since external fixation is a common procedure orthopaedic surgeons frequently do for trauma, and minimum expertise is required for surgeon eligibility.

#### Analysis methods

Full statistical analysis plan predefining all analyses and patient populations will be in place prior to any comparative analyses according to guidelines.^41^ KARDS will be reported according to the Consolidated Standards of Reporting Trials extension for Non-Inferiority and Equivalence Randomised Trials.^42^ The intention-to-treat (ITT) population will include all randomised participants, and the per-protocol (PP) population will include all participants who received their randomised intervention as intended. Although there is no ‘gold standard’ for non-inferiority trials, outcomes will be analysed primarily for the PP population.^43^ A sensitivity analysis will be conducted for the ITT population.

The primary analysis will report adjusted estimates of treatment effect from multivariable regression of KOOS pain score at 12 months. Statistical significance of KJD non-inferiority relative to KR will be based on a 2-sided likelihood-based test with type 1 error of 2.5% in both tails, adjusted by baseline score and OA severity as fixed effects, and delivery unit as a random effect.^44^ If the 95% confidence interval (CI) for absolute difference in means between KJD and KR lies entirely below or includes the non-inferiority boundary, then there would be insufficient evidence to reject the null hypothesis that KJD inferior to KR. Conversely, if the 95% CI lies entirely above the non-inferiority boundary, there would be evidence to reject the null hypothesis and conclude KJD non-inferior to KR. If non-inferiority demonstrated and KJD appears superior to KR, based on estimated effect and associated CI, statistical significance for superiority will be calculated based on an ITT analysis. Secondary analysis of the primary outcome measure will use multilevel modelling to account for longitudinal data collected over 24 months. Sensitivity analyses will be considered to investigate any impact of surgeon experience on treatment effect estimates.^45^

Reasons for missing data will be examined and primary method to account for missing data will be chosen based on the most reasonable missing data mechanism assumption, with sensitivity analyses to assess robustness of results to different missing data mechanism assumptions.

Other PROM responses will be transformed into dimension scores, according to scoring manuals, and presented graphically and longitudinally. Standardised area under the curve (AUC) statistics will be compared across treatment groups as an analysis conditional on patient time in the trial. Functional assessments will be reported descriptively, along with joint space width for the KJD group.

Complications will be reported as unique events and unique patients experiencing events. Joint survival will be measured from randomisation to time of further intervention and analysed using the Kaplan-Meier method.

Process evaluation interview data will be analysed using thematic content analysis to identify patterns or themes,^46^ using coding of audio-transcript recordings, adopting the framework method described by Ritchie and Spencer and Pope and Mays.^47 48^ Normalisation Process Theory will be used as a theoretical framework to explore and explain extent of intervention implementation,^49–51^ using the software package NVivo V.12 to manage data and facilitate this process. Interview data and full record of issues raised will be discussed in detail with the Trial Management Group and summarised for oversight committees. Good practice will be shared with other recruiting sites.

Cost-effectiveness analysis will be conducted from NHS and Personal Social Services perspectives and society over a 24-month time horizon. The analysis will estimate surgical intervention costs and primary and secondary healthcare services costs including complications, follow-up, medications and repeat medical procedures and out of pocket and productivity costs to patients and their families. Outcomes will be evaluated using QALYs estimated by the AUC approach. Unit costs will be obtained from list prices for devices and materials involved in the interventions, medications list prices, NHS health professional staff salary scales, primary care and community services opportunity costs,^52^ outpatient, inpatient admissions and Accident and Emergency visits NHS Reference Costs and median UK gross hourly earnings.^53^ Generalised linear models will be used to adjust for unbalanced baseline covariates in costs^54 55^ and adjusting for baseline EQ-5D-3L score in analysing QALYs.^56^ Missing data will be imputed using established methods.^57^ Results will be presented in terms of incremental cost per QALY gained and cost per unit gain in 12-month KOOS. Sampling uncertainty will be analysed using the bootstrap method^58^ and joint uncertainty in costs and QALYs will be analysed using cost-effectiveness acceptability curves.^59^

A decision analytic model will be built to evaluate lifetime cost-effectiveness over 20 years by adapting and updating a published Markov model of delayed joint replacement using National Joint Registry, clinical study and UK life table data.^9 10^ The model will account for trade-offs of delaying KR in terms of reducing the risk of the patient requiring revision surgery near end of life and increased complication risk with primary operation at older age.^9^ Sampling uncertainty in model parameter values will be described using probabilistic sensitivity analysis, while key parameters affecting the likelihood of KJD meeting the NICE £20 000 threshold for cost-effectiveness^60^ will be identified using Tornado plots.

### Monitoring

An independent Trial Steering Committee (TSC), comprising a statistician, two orthopaedic consultant surgeons and one patient representative, will have overall responsibility for trial oversight, monitoring trial progress, protocol adherence and participant safety. An independent Data Monitoring and Ethics Committee (DMEC) comprising a statistician and two orthopaedic consultant surgeons will review interim safety data by randomised group, reviewing the underlying statistical design assumptions to ensure the trial remains adequately powered. TSC and DMEC meetings will be conducted annually as a minimum according to agreed TSC and DMEC Charters.^61^

No formal guidelines for stopping the trial early are in place since no formal planned interim analysis of the primary outcome is planned.

Information on complications will be collected from randomisation to end of trial defined as the last visit date of the last patient. Serious complications will be subjected to expedited reporting where sites will inform CTRU within 24 hours of becoming aware of it. Suspected or confirmed pregnancies and all deaths from randomisation until the end of trial will be reported to CTRU.

## Ethics and dissemination

KARDS is funded by NIHR HTA (reference: 17/122/06) and sponsored by the University of Leeds, approved by the Research Ethics Committee (REC) (reference: 19/YH/0368) and Health Research Authority (HRA). All amendments will be submitted for approval and communicated to sites in accordance with HRA guidelines.

Trial results will be disseminated at relevant clinical conferences and societies, published in peer-reviewed journals and disseminated through relevant patient groups. Authorship will be according to International Committee of Medical Journal Editors (ICMJE) guidelines.

## Discussion

KARDS is a pragmatic, multicentre prospective randomised controlled trial conducted in an NHS setting, the aim is to determine if KJD is non-inferior to KR in terms of pain and cost-effective in the NHS, then it could be routinely offered to patients aged 65 years or less. In addition, it will report on radiological outcomes and patient acceptability. It will be a definitive IDEAL stage 3 (Assessment) trial^21^ with potential to lead to a paradigm shift if it demonstrates non-inferiority of KJD compared with KR.

Joint distraction outcomes at various anatomical locations have been reported in several case series. Though small numbers of patients have been involved, results are encouraging in at least providing temporary symptom relief. At the ankle, improvements in reported symptoms were seen in 73%–91% of patients at mean follow-up time of 1–12 years.^62^ Joint distraction has been demonstrated to give good clinical outcomes in first carpometacarpal joint osteoarthritis, albeit in a very limited number of patients. Patients were followed for 1 year with improved functional scores compared with baseline.^63^ The KJD literature is difficult to assess due to heterogeneity of devices and methods used. A recent review included one cohort study and two small trials all of which came from the same research group, including a total of 62 patients.^64^ These studies all utilised a spring-loaded static distractor. Western Ontario and McMaster Universities Osteoarthritis Index score improvements were significantly greater 1 year post KJD than conservatively managed osteoarthritis,^17^ and not inferior to total KR^19^ or high tibial osteotomy (HTO).^11^

Two studies^11 19^ reported KOOS, Intermittent and Constant Osteoarthritis Pain score, EQ-5D and Short Form (SF)−36 with significant improvements at 1 year seen in all scores except for the SF-36 mental component score, with no significant difference in these improvements compared with KR or HTO. Pain score assessed on pain VAS was reported in both studies and showed improvements at 1 year with no significant difference between KJD and HTO or KR. Radiographic assessment of joint structure has been undertaken in various studies, with imaging at the time of distraction or follow-up. The group above used MRI to assess structural recovery. Mean cartilage thickness was shown to increase on both the tibial and femoral sides and percentage of joint surface appearing as denuded subchondral bone decreased.^64^ Radiographic minimum joint space width was shown to increase by 0.8 mm at 12 months compared with baseline.^11 17 19^ Similar to another study where the mean joint space width, measured using standardised digital techniques, increased from 2.7 mm to 3.6 mm 12 months post-fixator removal.^65^

The most frequently reported KJD complication is pin site infection. Rates approaching 70% have been reported, with 20% of affected patients requiring intravenous therapy.^64^ In the series of 62 patients described above, two patients required surgical intervention for pin-site infection during distraction, with a further case of osteomyelitis requiring surgery following fixator removal.^11 17 19^ These infection rates are at odds with those reported in patients treated by definitive external fixation for other reasons. Pin-site infection rates of 40% are found fairly consistently, even where fixators are in place for much longer, the reasons for this are unclear.^66^ While transient pin-site infection seldom has long-term implications, it is unpleasant for patients and may impair rehabilitation. Deep infections may be more worrisome, especially considering expected osteoarthritis progression following distraction potentially requiring eventual arthroplasty. Wherever possible, external fixator pins will be sited outside the implantation zone of a KR. Total KR following significant osteomyelitis is significantly more complex and has further infection risk even when infection considered eradicated.^67^ Current KJD literature does not provide sufficient evidence to estimate serious infection rates following conversion to KR. In one ankle distraction study with over 5-year follow-up, there was no infection seen in five patients who had conversion to arthroplasty.^68^ Loss of knee range of movement immediately following distraction therapy has been observed to return after 1 year, with a small number of patients undergoing joint manipulation under anaesthetic to achieve this.^13 19^

Trial strengths include its pragmatic nature, standardised surgical and assessment techniques, robust reporting and safety mechanisms and appropriate sample size. The window of 6 weeks between baseline measures and planned surgery date aligns with clinical pathways and ensures recruitment feasibility. KARDS has a pragmatic hybrid expertise-based design, where surgeons are categorised into ‘delivery units’ based on their experience, a successful approach in similar knee OA surgical trials.^69^ Furthermore, clinicians are free to choose KR implant type and KJD external fixator. This choice brings a limitation in not being able to determine potential individual mechanisms of action limiting individual indications and/or contraindications. Those implants and fixators approved in the trial protocol are based on consensus among experts and published literature. Subgroup analysis will not be adequately powered to determine if a particular fixator type is superior. A further limitation is the lack of blinding, but this is unavoidable. It would be impractical to blind medical staff prior to surgery at many sites as they need to plan for the specific surgery. PPI feedback was that being blinded until just before or after surgery would be unacceptable if the medical team knew the allocation. The primary outcome measure is patient reported and, therefore, it is not possible to have a blinded primary outcome assessment.

## Supplementary Material

Reviewer comments

Author's manuscript

## References

[1] Lawrence RC, Felson DT, Helmick CG, et al. Estimates of the prevalence of arthritis and other rheumatic conditions in the United States. Part II. Arthritis Rheum 2008;58:26–35. 10.1002/art.23176 18163497 PMC3266664

[2] Skou ST, Roos EM, Laursen MB, et al. A randomized, controlled trial of total knee replacement. N Engl J Med 2015;373:1597–606. 10.1056/NEJMoa1505467 26488691

[3] Bayliss LE, Culliford D, Monk AP, et al. The effect of patient age at intervention on risk of implant revision after total replacement of the hip or knee: a population-based cohort study. Lancet 2017;389:1424–30. 10.1016/S0140-6736(17)30059-4 28209371 PMC5522532

[4] Deehan DJ, Murray JD, Birdsall PD, et al. Quality of life after knee revision arthroplasty. Acta Orthop 2006;77:761–6. 10.1080/17453670610012953 17068707

[5] Stambough JB, Clohisy JC, Barrack RL, et al. Increased risk of failure following revision total knee replacement in patients aged 55 years and younger. Bone Joint J 2014;96-B:1657–62. 10.1302/0301-620X.96B12.34486 25452369

[6] James Lind Alliance. About the James Lind alliance. Available: http://www.jla.nihr.ac.uk/about-the-james-lind-alliance/ [Accessed 28 Jul 2018].

[7] Price AJ, Longino D, Rees J, et al. Are pain and function better measures of outcome than revision rates after TKR in the younger patient? Knee 2010;17:196–9. 10.1016/j.knee.2009.09.003 20133136

[8] Bitton R. The economic burden of osteoarthritis. Am J Manag Care 2009;15:S230–5. 19817509

[9] Mota REM. Cost-Effectiveness analysis of early versus late total hip replacement in Italy. Value Health 2013;16:267–79. 10.1016/j.jval.2012.10.020 23538178

[10] Lan RH, Yu J, Samuel LT, et al. How are we measuring cost-effectiveness in total joint arthroplasty studies? systematic review of the literature. J Arthroplasty 2020;35:3364–74. 10.1016/j.arth.2020.06.046 32680755

[11] van der Woude JAD, Wiegant K, van Heerwaarden RJ, et al. Knee joint distraction compared with high tibial osteotomy: a randomized controlled trial. Knee Surg Sports Traumatol Arthrosc 2017;25:876–86. 10.1007/s00167-016-4131-0 27106926 PMC5332499

[12] Jansen MP, Besselink NJ, van Heerwaarden RJ, et al. Knee joint distraction compared with high tibial osteotomy and total knee arthroplasty: two-year clinical, radiographic, and biochemical marker outcomes of two randomized controlled trials. Cartilage 2021;12:181–91. 10.1177/1947603519828432 30758214 PMC7970375

[13] Jansen MP, Mastbergen SC, van Heerwaarden RJ, et al. Knee joint distraction in regular care for treatment of knee osteoarthritis: a comparison with clinical trial data. PLoS One 2020;15:e0227975. 10.1371/journal.pone.0227975 31968005 PMC6975543

[14] Wiegant K, van Roermund PM, Intema F, et al. Sustained clinical and structural benefit after joint distraction in the treatment of severe knee osteoarthritis. Osteoarthritis Cartilage 2013;21:1660–7. 10.1016/j.joca.2013.08.006 23954704

[15] Bellamy N, Buchanan WW, Goldsmith CH. Validation study of WOMAC: a health status instrument for measuring clinically important patient relevant outcomes to antirheumatic drug therapy in patients with osteoarthritis of the hip or knee. J Rheumatol 1988;15:1833–40. 3068365

[16] Lequesne M. Indices of severity and disease activity for osteoarthritis. Semin Arthritis Rheum 1991;20:48–54. 10.1016/0049-0172(91)90027-w 1866630

[17] van der Woude J-TAD, Wiegant K, van Roermund PM, et al. Five-Year follow-up of knee joint distraction: clinical benefit and cartilaginous tissue repair in an open uncontrolled prospective study. Cartilage 2017;8:263–71. 10.1177/1947603516665442 28618871 PMC5625862

[18] van der Woude JAD, Wiegant K, van Heerwaarden RJ, et al. Knee joint distraction compared with total knee arthroplasty. Bone Joint J 2017;99-B:51–8. 10.1302/0301-620X.99B1.BJJ-2016-0099.R3 28053257

[19] van der Woude JAD, Nair SC, Custers RJH, et al. Knee joint distraction compared to total knee arthroplasty for treatment of end stage osteoarthritis: simulating long-term outcomes and cost-effectiveness. PLoS One 2016;11:e0155524. 10.1371/journal.pone.0155524 27171268 PMC4865158

[20] Wiegant K, Intema F, van Roermund PM, et al. Evidence of cartilage repair by joint distraction in a canine model of osteoarthritis. Arthritis Rheumatol 2015;67:465–74. 10.1002/art.38906 25303046

[21] Hirst A, Philippou Y, Blazeby J, et al. No surgical innovation without evaluation. Ann Surg 2019;269:211–20. 10.1097/SLA.0000000000002794 29697448

[22] Blencowe NS, Mills N, Cook JA, et al. Standardizing and monitoring the delivery of surgical interventions in randomized clinical trials. Br J Surg 2016;103:1377–84. 10.1002/bjs.10254 27462835 PMC5132147

[23] Kellgren JH, Lawrence JS. Radiological assessment of osteo-arthrosis. Ann Rheum Dis 1957;16:494–502. 10.1136/ard.16.4.494 13498604 PMC1006995

[24] Chan A-W, Tetzlaff JM, Gøtzsche PC, et al. Spirit 2013 explanation and elaboration: guidance for protocols of clinical trials. BMJ 2013;346:e7586-e. 10.1136/bmj.e7586 23303884 PMC3541470

[25] Rolfson O, Wissig S, van Maasakkers L, et al. Defining an international standard set of outcome measures for patients with hip or knee osteoarthritis: consensus of the International Consortium for health outcomes measurement hip and knee osteoarthritis Working group. Arthritis Care Res 2016;68:1631–9. 10.1002/acr.22868 PMC5129496 26881821

[26] Hawker GA, Mian S, Kendzerska T, et al. Measures of adult pain: visual analog scale for pain (vas pain), numeric rating scale for pain (NRS pain), McGill pain questionnaire (MPQ), short-form McGill pain questionnaire (SF-MPQ), chronic pain grade scale (CpGs), short Form-36 bodily pain scale (SF-36 BPs), and measure of intermittent and constant osteoarthritis pain (ICOAP). Arthritis Care Res 2011;63 Suppl 11:S240–52. 10.1002/acr.20543 22588748

[27] McCormack HM, Horne DJ, Sheather S. Clinical applications of visual analogue scales: a critical review. Psychol Med 1988;18:1007–19. 10.1017/s0033291700009934 3078045

[28] Dawson J, Fitzpatrick R, Murray D, et al. Questionnaire on the perceptions of patients about total knee replacement. J Bone Joint Surg Br 1998;80:63–9. 10.1302/0301-620x.80b1.7859 9460955

[29] Dunbar MJ, Robertsson O, Ryd L, et al. Appropriate questionnaires for knee arthroplasty. Results of a survey of 3600 patients from the Swedish knee arthroplasty registry. J Bone Joint Surg Br 2001;83:339–44. 10.1302/0301-620x.83b3.11134 11341416

[30] Xie F, Ye H, Zhang Y, et al. Extension from inpatients to outpatients: validity and reliability of the Oxford knee score in measuring health outcomes in patients with knee osteoarthritis. Int J Rheum Dis 2011;14:206–10. 10.1111/j.1756-185X.2010.01580.x 21518321

[31] CDC. Timed Up & Go. Available: https://www.cdc.gov/steadi/pdf/TUG_test-print.pdf

[32] Davis AM, King LK, Stanaitis I. Fundamentals of osteoarthritis: outcome evaluation with patient-reported measures and functional tests, 2021. 10.1016/j.joca.2021.07.016 34534660

[33] GOV.UK. Surgical site infection surveillance service: protocol and guides [Internet]. Available: https://www.gov.uk/government/publications/surgical-site-infection-surveillance-service-protocol-procedure-codes-and-user-manual [Accessed 09 Sep 2019].

[34] Marijnissen ACA, Vincken KL, Vos PAJM, et al. Knee images digital analysis (KIDA): a novel method to quantify individual radiographic features of knee osteoarthritis in detail. Osteoarthritis Cartilage 2008;16:234–43. 10.1016/j.joca.2007.06.009 17693099

[35] W-Dahl A, Toksvig-Larsen S, Roos EM. Association between knee alignment and knee pain in patients surgically treated for medial knee osteoarthritis by high tibial osteotomy. A one year follow-up study. BMC Musculoskelet Disord 2009;10:154. 10.1186/1471-2474-10-154 19995425 PMC2796991

[36] Nilsdotter AK, Toksvig-Larsen S, Roos EM. Knee arthroplasty: are patients' expectations fulfilled? A prospective study of pain and function in 102 patients with 5-year follow-up. Acta Orthop 2009;80:55–61. 10.1080/17453670902805007 19234886 PMC2823230

[37] Roos EM, Toksvig-Larsen S. Knee injury and Osteoarthritis Outcome Score (KOOS) - validation and comparison to the WOMAC in total knee replacement. Health Qual Life Outcomes 2003;1:17. 10.1186/1477-7525-1-17 12801417 PMC161802

[38] Arthritis Research UK. State of musculoskeletal health 2017, 2017.

[39] Collins NJ, Misra D, Felson DT, et al. Measures of knee function: international knee documentation Committee (IKDC) subjective knee evaluation form, knee injury and osteoarthritis outcome score (KOOS), knee injury and osteoarthritis outcome score physical function short form (KOOS-PS), knee outcome survey activities of daily living scale (KOS-ADL), Lysholm knee scoring scale, Oxford knee score (OKS), Western Ontario and McMaster universities osteoarthritis index (WOMAC), activity rating scale (ARS), and Tegner activity score (Tas). Arthritis Care Res 2011;63 Suppl 11:S208–28. 10.1002/acr.20632 PMC4336550 22588746

[40] Devji T, Guyatt GH, Lytvyn L, et al. Application of minimal important differences in degenerative knee disease outcomes: a systematic review and case study to inform BMJ Rapid Recommendations. BMJ Open 2017;7:e015587. 10.1136/bmjopen-2016-015587 PMC5777462 28495818

[41] Gamble C, Krishan A, Stocken D, et al. Guidelines for the content of statistical analysis plans in clinical trials. JAMA 2017;318:2337–43. 10.1001/jama.2017.18556 29260229

[42] Piaggio G, Elbourne DR, Pocock SJ, et al. Reporting of noninferiority and equivalence randomized trials: extension of the CONSORT 2010 statement. JAMA 2012;308:2594–604. 10.1001/jama.2012.87802 23268518

[43] Rehal S, Morris TP, Fielding K, et al. Non-Inferiority trials: are they inferior? A systematic review of reporting in major medical journals. BMJ Open 2016;6:e012594. 10.1136/bmjopen-2016-012594 PMC5073571 27855102

[44] Beard DJ, Davies LJ, Cook JA, et al. The clinical and cost-effectiveness of total versus partial knee replacement in patients with medial compartment osteoarthritis (TOPKAT): 5-year outcomes of a randomised controlled trial. Lancet 2019;394:746–56. 10.1016/S0140-6736(19)31281-4 31326135 PMC6727069

[45] Corrigan N, Marshall H, Croft J, et al. Exploring and adjusting for potential learning effects in ROLARR: a randomised controlled trial comparing robotic-assisted vs. standard laparoscopic surgery for rectal cancer resection. Trials 2018;19:339. 10.1186/s13063-018-2726-0 29945673 PMC6020359

[46] Braun V, Clarke V. Using thematic analysis in psychology. Qual Res Psychol 2006;3:77–101. 10.1191/1478088706qp063oa

[47] Ritchie J, Spencer L. Qualitative data analysis for applied policy research. In: Bryman A, Burgess RG, eds. Analyzing Qualitative Data.. London, UK: Routledge, 1994.

[48] Pope C, Mays N. Reaching the parts other methods cannot reach: an introduction to qualitative methods in health and health services research. BMJ 1995;311:42–5. 10.1136/bmj.311.6996.42 7613329 PMC2550091

[49] May C, Finch T. Implementing, embedding, and integrating practices: an outline of normalization process theory. Sociology 2009;43:535–54. 10.1177/0038038509103208

[50] May C, Finch T, Mair F, et al. Understanding the implementation of complex interventions in health care: the normalization process model. BMC Health Serv Res 2007;7:148. 10.1186/1472-6963-7-148 17880693 PMC2089069

[51] May CR, Mair F, Finch T, et al. Development of a theory of implementation and integration: normalization process theory. Implement Sci 2009;4:29. 10.1186/1748-5908-4-29 19460163 PMC2693517

[52] Curtis L, Burns A. Unit costs of health and social care 2020, personal social services research unit. Canterbury: University of Kent, 2020.

[53] Statistics OfN. Annual survey of hours and earnings (ASHE), 2020. Available: https://www.ons.gov.uk/employmentandlabourmarket/peopleinwork/earningsandworkinghours/

[54] Mihaylova B, Briggs A, O'Hagan A, et al. Review of statistical methods for analysing healthcare resources and costs. Health Econ 2011;20:897–916. 10.1002/hec.1653 20799344 PMC3470917

[55] Thompson SG, Nixon RM. How sensitive are cost-effectiveness analyses to choice of parametric distributions? Med Decis Making 2005;25:416–23. 10.1177/0272989X05276862 16061893

[56] Manca A, Hawkins N, Sculpher MJ. Estimating mean QALYs in trial-based cost-effectiveness analysis: the importance of controlling for baseline utility. Health Econ 2005;14:487–96. 10.1002/hec.944 15497198

[57] Faria R, Gomes M, Epstein D, et al. A guide to handling missing data in cost-effectiveness analysis conducted within randomised controlled trials. Pharmacoeconomics 2014;32:1157–70. 10.1007/s40273-014-0193-3 25069632 PMC4244574

[58] Briggs AH, Wonderling DE, Mooney CZ. Pulling cost-effectiveness analysis up by its bootstraps: a non-parametric approach to confidence interval estimation. Health Econ 1997;6:327–40. 10.1002/(sici)1099-1050(199707)6:4&lt;327::aid-hec282&gt;3.0.co;2-w 9285227

[59] Fenwick E, Claxton K, Sculpher M. Representing uncertainty: the role of cost-effectiveness acceptability curves. Health Econ 2001;10:779–87. 10.1002/hec.635 11747057

[60] National Institute for H, Care E. NICE Process and Methods Guides. Guide to the methods of technology appraisal 2013. London: National Institute for health and care excellence (NICE) Copyright © 2013 National Institute for health and clinical excellence, unless otherwise stated. all rights reserved, 2013.

[61] DAMOCLES Study Group, NHS Health Technology Assessment Programme. A proposed charter for clinical trial data monitoring committees: helping them to do their job well. Lancet 2005;365:711–22. 10.1016/S0140-6736(05)17965-3 15721478

[62] van Valburg AA, van Roermund PM, Lammens J, et al. Can Ilizarov joint distraction delay the need for an arthrodesis of the ankle? A preliminary report. J Bone Joint Surg Br 1995;77:720–5. 10.1302/0301-620X.77B5.7559696 7559696

[63] Spaans AJ, Minnen LPvan, Braakenburg A, et al. Joint distraction for thumb carpometacarpal osteoarthritis: a feasibility study with 1-year follow-up. J Plast Surg Hand Surg 2017;51:254–8. 10.1080/2000656X.2016.1241789 27758127

[64] Takahashi T, Baboolal TG, Lamb J, et al. Is Knee Joint Distraction a Viable Treatment Option for Knee OA?-A Literature Review and Meta-Analysis. J Knee Surg 2019;32:788–95. 10.1055/s-0038-1669447 30157528

[65] Intema F, Van Roermund PM, Marijnissen ACA, et al. Tissue structure modification in knee osteoarthritis by use of joint distraction: an open 1-year pilot study. Ann Rheum Dis 2011;70:1441–6. 10.1136/ard.2010.142364 21565898 PMC3128325

[66] Ferguson D, Harwood P, Allgar V, et al. The pins trial: a prospective randomized clinical trial comparing a traditional versus an emollient skincare regimen for the care of pin-sites in patients with circular frames. Bone Joint J 2021;103-B:279–85. 10.1302/0301-620X.103B2.BJJ-2020-0680.R1 33517738

[67] Williams RL, Khan W, Roberts-Huntleigh N, et al. Total knee arthroplasty in patients with prior adjacent multi-organism osteomyelitis. Acta Orthop Belg 2018;84:184–91. 30462602

[68] Nguyen MP, Pedersen DR, Gao Y, et al. Intermediate-Term follow-up after ankle distraction for treatment of end-stage osteoarthritis. J Bone Joint Surg Am 2015;97:590–6. 10.2106/JBJS.N.00901 25834084 PMC4372990

[69] Beard D, Price A, Cook J, et al. Total or Partial Knee Arthroplasty Trial - TOPKAT: study protocol for a randomised controlled trial. Trials 2013;14:292. 10.1186/1745-6215-14-292 24028414 PMC3848560

