# Supplementary material for: Clinical and cost-effectiveness of Knee Arthroplasty versus Joint Distraction for Osteoarthritis (KARDS): protocol for a multicentre, phase III, randomised control trial
Source: BMJ Open. 2022 Jun 30;12(6):e062721. doi: 10.1136/bmjopen-2022-062721 (PMC9247693; doi:10.1136/bmjopen-2022-062721)
Supplement: Supplementary data [file bmjopen-2022-062721supp001.pdf]

	**Screening and Consent**	**Randomisation & Baseline**		**Surgery (Day 0)**	**Follow-up time point**						**Unscheduled**
					**Clinic visits**			**Postal questionnaire packs**	**Clinic visits**		
	Prior to registration	Before randomisation and within 6 weeks prior to surgery	After randomisation and within 6 weeks prior to surgery		Post-operative (Up to Day 7)	Fixator Removal (Week 6)	Follow up (Month 3)	Follow up (Month 6)	Follow up (Month 12)	Follow up (Month 24)	
	All	All	All	All	All	KJD arm only	All	All	All	All	All
Informed Consent	X										
Screening Data	X										
Eligibility	X										
Patient Details	X										
Patient Demographics		X									
Medical History		X									
OA Severity (Kellgren-Lawrence grade based on standard AP & lateral x-rays)		X									
Physical examination of knee		X									
TUG (Timed up and go test)		X					X		X	X	
ROM (Range of movement) using goniometer		X					X		X	X	
Rosenberg View X-ray			X				X^		X^	X^	
Surgery (KR or KJD)				X							
Surgical details				X		X^					
Distraction of external fixator (KJD only)				X^	X^						
Removal of external fixator (KJD only)						X^					
Intra-operative Complications				X		X^					
Additional knee related and/or other limb surgery				X	X	X^	X	X	X	X	X
Concomitant Medications				X	X	X^	X	X	X	X	X
Discharge Details					X	X^					

AP/Lateral View X-rays					X	X^					
Post-operative Complications					X	X^	X	X	X	X	X
**Patient Reported Outcomes**											
KOOS		X		X*			X	X	X	X	
OKS		X					X	X	X	X	
EQ5D-3L		X					X	X	X	X	
Pain VAS		X					X	X	X	X	
Health Resource Use		X					X	X	X	X	
Serious complications											X
Participant withdrawal											X
Re-operation											X
Pregnancy											X
Death											X

*Up to 1 day before surgery

^KJD arm only
