# Supplementary material for: Clinical and cost-effectiveness of Knee Arthroplasty versus Joint Distraction for Osteoarthritis (KARDS): protocol for a multicentre, phase III, randomised control trial
Source: BMJ Open. 2022 Jun 30;12(6):e062721. doi: 10.1136/bmjopen-2022-062721 (PMC9247693; doi:10.1136/bmjopen-2022-062721)
Supplement: Author's manuscript [file bmjopen-2022-062721.draft_revisions.pdf]

BMJ Open is committed to open peer review. As part of this commitment we make the peer review history of every article we publish publicly available.

When an article is published we post the peer reviewers' comments and the authors' responses online. We also post the versions of the paper that were used during peer review. These are the versions that the peer review comments apply to.

The versions of the paper that follow are the versions that were submitted during the peer review process. They are not the versions of record or the final published versions. They should not be cited or distributed as the published version of this manuscript.

BMJ Open is an open access journal and the full, final, typeset and author-corrected version of record of the manuscript is available on our site with no access controls, subscription charges or pay-per-view fees (http://bmjopen.bmj.com).

If you have any questions on BMJ Open's open peer review process please email info.bmjopen@bmj.com

BMJ Open

Clinical and Cost-effectiveness of Knee Arthroplasty versus Joint Distraction for Osteoarthritis (KARDS): Protocol for A Multicentre, Phase III, Randomised Control Trial

Journal:	*BMJ Open*
Manuscript ID	bmjopen-2022-062721
Article Type:	Protocol
Date Submitted by the Author:	14-Mar-2022

Complete List of Authors:	Tassinari, Cerys; University of Leeds Leeds Institute of Clinical Trials Research, Statistics<br>Higham, Ruchi; University of Leeds Leeds Institute of Clinical Trials Research, Trial Management<br>Smith, Isabelle; University of Leeds Clinical Trials Research Unit,<br>Arnold, Susanne; University of Warwick, ; The University of Warwick<br>Mujica-Mota, Ruben; University of Leeds, Leeds Institute of Health Sciences<br>Metcalfe, Andrew; University of Warwick Warwick Medical School, Warwick Clinical Trials Unit; University Hospitals Coventry and Warwickshire NHS Trust, Trauma and Orthopaedics<br>Simpson, Hamish; University of Edinburgh,<br>Murray, David; University of Oxford Nuffield Department of Orthopaedics Rheumatology and Musculoskeletal Sciences<br>McGonagle, Dennis; 1. Leeds Institute of Rheumatic and Musculoskeletal Medicine, University of Leeds, NIHR Leeds Biomedical Research Centre, Chapel Allerton Hospital<br>Sharma, Hemant; Hull and East Yorkshire Hospitals NHS Trust, Department of Orthopaedics<br>Hamilton, Thomas; University of Oxford, NDORMS<br>Ellard, David; University of Warwick, Clinical Trials Unit;<br>Fernandez, Catherine; University of Leeds Faculty of Medicine and Health, Clinical Trials Research Unit<br>Reynolds, Catherine; University of Leeds, Clinical Trials Research Unit<br>Harwood, Paul; University of Leeds Leeds Institute of Medical Research<br>Croft, Julie; University of Leeds, Clinical Trials Research Unit, Leeds Institute of Clinical Trials<br>Stocken, Deborah; University of Leeds Leeds Institute of Clinical Trials Research, Statistics<br>Pandit, Hemant ; Chapel Allerton Hospital; 1. Leeds Institute of Rheumatic and Musculoskeletal Medicine, University of Leeds, NIHR Leeds Biomedical Research Centre, Chapel Allerton Hospital

Keywords:	Knee < ORTHOPAEDIC & TRAUMA SURGERY, Musculoskeletal disorders < ORTHOPAEDIC & TRAUMA SURGERY, Orthopaedic & trauma surgery < SURGERY, STATISTICS & RESEARCH METHODS

Clinical and Cost-effectiveness of Knee Arthroplasty versus Joint Distraction for Osteoarthritis (KARDS): Protocol for A Multicentre, Phase III, Randomised Control Trial

Authors and affiliations

Cerys Tassinari[1*], Ruchi Higham[1*], Isabelle Smith[1], Susanne Arnold[2], Ruben Mujica-Mota[3], Andy Metcalfe[2], A Hamish RW Simpson[4], David Murray[5], Dennis McGonagle[6], Hemant K Sharma[7], Thomas W Hamilton[5], David R Ellard[2], Catherine Fernandez[1], Catherine Reynolds [1], Paul Harwood[8], Julie Croft[1], Deborah D Stocken[1^], Hemant Pandit[6^]

Acknowledgements

Roberta Longo[3]

---

* Joint First Authors

^ Joint Senior Authors

[1] Clinical Trials Research Unit, University of Leeds, UK

[2] Warwick Clinical Trials Unit, University of Warwick, UK

[3] Leeds Institute of Health Sciences, University of Leeds, UK

[4] Department of Orthopaedics and Trauma, University of Edinburgh, UK

[5] Nuffield Department of Orthopaedics Rheumatology and Musculoskeletal Science, University of Oxford, UK

[6] Leeds Institute of Rheumatic and Musculoskeletal Medicine, University of Leeds, UK

[7] Department of Trauma and Orthopaedics, Hull University Teaching Hospitals, Hull, UK

[8] Leeds Institute of Medical Research, University of Leeds, UK

Corresponding author

Cerys Tassinari; C.Tassinari@leeds.ac.uk

Word Count: (excluding title, abstract, references, tables & figures) 4102(Limit: 4000)

Abstract Word Count: 300 (Limit: 300)

For peer review only

ABSTRACT

Introduction

Knee Replacement (KR) is a clinically-proven procedure typically offered to patients with severe knee Osteoarthritis (OA) to relieve pain and improve quality of life. However, artificial joints fail over time, requiring revision associated with higher mortality and inferior outcomes. With more young people presenting with knee OA and increasing life expectancy, there is an unmet need to postpone time to first KR. Knee Joint Distraction (KJD), the practice of using external fixators to open up knee joint space, is proposed as potentially effective to preserve the joint following initial studies in the Netherlands, however, has not been researched within an NHS setting. The KARDS trial will investigate whether KJD is non-inferior to KR in terms of patient-reported post-operative pain 12 months post-surgery.

Methods and analysis

KARDS is a phase III, multi-centre, pragmatic, open-label, individually randomised controlled non-inferiority trial comparing KJD with KR in patients with severe knee OA, employing a hybrid-expertise design, with internal pilot phase and process evaluation. 344 participants will be randomised (1:1) to KJD or KR. The primary outcome measure is the Knee Injury and Osteoarthritis Outcomes Score (KOOS) pain domain score at 12-months post-operation. Secondary outcome measures include patient reported overall KOOS, Pain VAS and Oxford Knee Scores, knee function assessments, joint space width, complications and further interventions over 24 months post-operation. Per patient cost difference between KR and KJD and cost per QALY gained over 24 months will be estimated within trial, and incremental cost per QALY gained over 20 years by KJD relative to KR predicted using decision analytic modelling.

Ethics and dissemination

Ethics approval was obtained from the Research Ethics Committee (REC) and Health Research Authority (HRA). Trial results will be disseminated at clinical conferences, through relevant patient groups and published in peer-reviewed journals.

Trial registration number: ISRCTN14879004. Recruitment opened April 2021.

For peer review only

Strengths and limitations of this trial

- The KARDS trial is a pragmatic trial design using standardised surgical and assessment techniques, robust reporting and safety mechanisms and appropriate sample size.

- A hybrid-expertise based design has been adopted to ensure feasibility of the trial whilst accounting for surgeon experience and potential lack of individual equipoise.

- A surgical manual will document each trial procedure highlighting mandatory components according to recommended guidance for surgery trials. This will allow comprehensive reporting of the interventions delivered during the trial.

- Due to the nature of the interventions, the trial personnel and participants are not blinded to treatment allocation.

MAIN TEXT

Background

Osteoarthritis (OA) is the commonest musculoskeletal condition that affects joints, causing pain, joint dysfunction and significant quality of life (QoL) impact. With rising obesity rates and ageing population, the number of people presenting with knee OA is increasing (1).

Patients with severe knee OA experiencing joint symptoms that substantially impact QoL are typically offered knee replacement (KR) to relieve pain and improve mobility. KR is clinically proven and cost-effective (2), however, artificial joints have a finite life span. If KR fails, revision is complex, costly and associated with higher morbidity, mortality, and inferior outcomes (3-5).

The James Lind Alliance established that defining the optimum timing of joint replacement, in order to achieve the best outcome is a significant patient concern(6). The number of young patients (55 years or less) undergoing KR is increasing (5), and risk of failure is disproportionately higher in the young and active. A combined endpoint analysis including revision, poor function and significant pain has shown KR success to be as low as 59% after 12-years in patients 60 years or less (7). Increasing life expectancy and the growing number of younger patients means there is a need for treatment which postpones the time to first KR, without compromising QoL or hampering ability to undergo KR at a later stage (7, 8). As in joint replacement in general, it is unknown whether treatment options preserving the joint are cost-effective (9, 10).

Knee Joint Distraction (KJD), the practice of placing an external fixator across a synovial joint and pulling the joint surfaces apart approximately 5mm for ~6 weeks, has been proposed as a potentially effective alternative to preserve the joint. The aim is to harness intrinsic joint-repair potential, providing cartilage repair and normalisation of subchondral bone abnormalities (11). KJD is not currently widely used in the UK, and no trials have been conducted in the NHS. Initial studies conducted in the Netherlands suggest it a safe and potentially effective treatment (12-14)One small trial suggested KJD to be non-inferior to total KR in function (15-17) and another predicted that it could save over 30% of revision KRs (18). With a willingness to pay €20,000 per Quality Adjusted Life Year (QALY), KJD was shown to be cost effective in over 75% cases for all age groups and over 90% in the young (55 years or less) (19).

For peer review only

Rationale

There is strong scientific basis for KJD with excellent cartilage regeneration in experimental OA with joint offloading procedures (20). Given the preliminary clinical data and underpinning science, KJD could be an alternative therapy to KR for younger patients, but current evidence is limited. Patient feedback highlighted the key priority for those in this age group is retaining their own knee, at expense of some residual knee pain. If KJD is shown to be safe, non-inferior to KR in terms of pain and cost-effective in the NHS then it could be routinely offered to patients 65 years or less, delaying need for KR and potentially avoiding revision surgery. This is the aim of the KARDS trial.

Methods and Design

Objectives

The primary objective is to conduct a multi-centre trial to investigate clinical effectiveness of KJD compared to KR in patients aged 65 years or less, with symptomatic knee OA severe enough to warrant KR, based on patient reported pain 12 months after surgery.

Secondary objectives are to investigate: 1) Patient reported outcomes; 2) Clinical outcomes of knee function; 3) Complications and need for further intervention; 4) Cost-effectiveness; 5) Participant experiences, intervention fidelity and barriers to wider implementation.

Trial design

This publication describes KARDS protocol V2.0, dated 29th September 2020.

KARDS is a phase III IDEAL Stage 3 Assessment (21), multi-centre, pragmatic, open-label, 1:1, two-arm individually randomised controlled trial, with embedded 12-month internal pilot phase.

The internal pilot phase will incorporate a qualitative process evaluation to identify potential barriers to recruitment and any challenges experienced in maintaining intervention fidelity. As part of the process evaluation, qualitative semi-structured interviews will be undertaken with clinicians, trial staff and participants to explore experiences of trial involvement and intervention acceptability during the pilot phase and throughout the main trial. Progression at the end of the pilot phase will be based on i) recruitment and dropout rates ii); safety; iii) the process evaluation.

<u>Trial setting and recruitment</u>

Participants will be recruited from secondary care orthopaedic centres following GP or specialist referral. Potentially eligible participants will be identified by the attending clinical team from orthopaedic outpatient

For peer review only

clinics and theatre lists. Following information provision, patients will be given the opportunity to discuss the trial with their family, friends and healthcare professionals before being invited to participate.

Informed consent will be obtained by the Principal Investigator (PI) or appropriate, delegated, healthcare professional as detailed on the Authorised Personnel Log, in accordance with the principles of Good Clinical Practice (GCP) and Declaration of Helsinki 1996.

All sites must be able to deliver both KR and KJD. As KJD is not a standard technique used in knee surgery not all surgeons will have the required experience, and some surgeons may not be in individual equipoise despite there being centre equipoise. A hybrid expertise-based design, where surgeons are categorised into “delivery units” based on experience, addresses both issues.

There are two delivery unit categories based on the interventions surgeons are authorised to perform within the trial: i) *Single delivery units* consist of surgeons authorised to deliver KJD **or** KR, where the surgeon performing the procedure will be chosen after randomisation, depending on the allocation, or ii) *Dual delivery unit*s, consisting surgeons authorised to deliver KJD **and** KR where a randomised participant may receive either operation by the same surgeon.

Eligibility

Surgeon eligibility: Participating surgeons must either be a consultant orthopaedic surgeon or perform the procedure under direct consultant supervision. To deliver KR within KARDS a surgeon must have performed ≥10 KRs in the past 12 months as the primary surgeon. To deliver KJD within KARDS they must have performed ≥10 external fixations during their career as the primary surgeon or completed a limb reconstruction fellowship.

Patient eligibility: criteria are minimised to ensure inclusivity and generalisability. Adult patients are eligible if aged ≤65 years requiring KR and meet the criteria in Table 1.

For peer review only

Table 1: Patient Eligibility Criteria

Inclusion Criteria	Exclusion Criteria
Age ≥18 years and ≤65 years at time of signing the Informed Consent form	Bone density not sufficient to support pins for 6 weeks*
Symptoms (pain and/or reduced function) severe enough to warrant knee replacement*	Isolated patella-femoral OA*
Pre-operative leg alignment not requiring correction*	Complete joint space obliteration in both medial and lateral tibio-femoral compartments as seen on weight bearing AP knee radiograph
Intact collateral knee ligaments*	A known diagnosis of inflammatory arthritis
Fixed flexion deformity ≤10[illegible]	Presence of a previous joint replacement in any limb
	Surgical treatment of involved knee within the past 6 months (excluding arthroscopy)
	Previous knee joint distraction on the involved knee
	Previously participated in the KARDS trial
	Weight >120kg
	Pregnant or lactating (confirmed by participant)
	Active cancer (currently diagnosed and under treatment)
	Unable to complete all trial procedures (e.g. attend follow up visits, complete questionnaires)
	Unable to provide informed consent (cognitive disorder such as dementia, psychiatric illness)

** In the opinion of the treating clinician*

<u>Interventions</u>

A surgical manual will document each trial procedure highlighting mandatory components according to recommended guidance (22).

Intervention (Knee Joint Distraction)

A definitive external fixator construct will be used which allows for controlled linear distraction across the knee joint of 5mm. The exact nature of the construct will depend on equipment availability at site and surgeon preference. Devices will be approved for trial use by the Trial Management Group

During surgery the external fixation frame will be assembled according to frame construct procedures detailed in the surgical manual, with focus on meticulous pin insertion to minimise complication risk. Pins will be placed under fluoroscopic control. Once assembly complete, ≥2mm and ≤5mm axial distraction will be applied across

For peer review only

the knee joint. A further 1mm distraction may be applied per day until 5mm distraction at the joint is confirmed radiographically, or up to 7 days.

External fixators will be removed under general or regional anaesthesia after six weeks. Local protocol for pin-site care will be followed and will be documented. Gentle manipulation under anaesthesia to achieve ≥90 degrees of motion will be attempted at the time of fixator removal.

Control (Knee replacement)

KR surgery will be performed in line with local practice and the surgical manual and will vary depending upon implant type and surgeon preference. Surgeons performing the procedure are expected to comply with specific surgical steps for the implant being used as detailed in the manufacturer instructions for use document.

Concomitant care and interventions

Pre-operative preparation and post-operative care will be provided to all trial participants in line with the site's usual protocol for KRs. Decisions about concomitant medications/treatments for symptomatic knee osteoarthritis will be according to local medical plan and clinical management. Details of analgesia and other medication prescribed will be collected throughout trial. Participants may require further intervention for symptomatic knee OA as per routine practice. Further clinical intervention is permitted for all participants and recorded for the trial.

Patient and Public Involvement

KARDS Patient and Public Involvement (PPI) group provided feedback on choice of primary outcome, minimally important difference used sample size calculations & the decision to not blind participants. PPI representatives on the Trial Management Group provided feedback on the schedule of events for participants.

Randomisation and blinding

Participants will be randomised into the trial by an authorised member of site staff, on a 1:1 basis between KJD and KR, based on a minimisation algorithm with random component balanced for delivery unit and OA severity (Kellgren-Lawrence Grades 2-3 vs. Grade 4) (23). Randomisation will be performed centrally using Leeds Clinical Trials Research Unit (CTRU) automated secure 24-hour randomisation web or telephone service, occurring on the same day as baseline visit, within 6 weeks of the planned surgery date. Clinical assessments and baseline questionnaires will be completed before randomisation with trial specific assessments performed afterwards. Treatment allocation will not be blinded to participants, medical staff, or clinical trial staff.

For peer review only

Data collection

Clinical data will be collected at baseline, day of surgery, prior to discharge, week 6 (KJD only), and months 3, 12, and 24 post-surgery. Participant completed data will be collected at baseline, day of surgery and months 3, 6, 12, and 24 post-surgery. Full assessment schedule based on the SPIRIT guidance (24) provided in Supplementary Material 1.

Participating sites will maintain a file of essential trial documentation including copies of all completed CRFs. Sites will post paper CRFs, and electronically transfer trial X-rays to Leeds CTRU. Trial data will be entered onto an electronic database, except post-surgery questionnaires completed using electronic remote data capture by participants or via postal questionnaire.

Data will be monitored for quality and completeness by CTRU. Missing data will be requested from sites until received, confirmed as unavailable or trial analysis begins. The sponsor reserves the right to conduct periodic source data verification to monitor trial integrity.

Participant qualitative interviews will be conducted by telephone, and staff interviews conducted in person, or telephone/video conference. Interviews will be audio recorded on an encrypted recorder, anonymised and transcribed verbatim for analysis.

All information collected during the trial will be kept strictly confidential. Information will be held securely on paper and electronically at Leeds CTRU, with process evaluation data held securely on Warwick CTU server. Both will comply with all aspects of the Data Protection Act 2018. If a participant withdraws consent from further trial treatment and/or further data collection, data to the point of withdrawal will remain on file and included in the analysis.

Outcome measures

Primary outcome measure

The primary outcome measure is Knee Injury and Osteoarthritis Outcomes Score (KOOS) pain score 12-months post-surgery. Pain was indicated by the PPI group as being the most important outcome to them. KOOS is a patient-administered questionnaire, validated for use in patients with knee OA or knee injury (25), recorded on a Likert Scale 0-4, transformed to 0 (worst) to 100 (best) scale.

Secondary outcome measures

1. *Patient report outcome measures (PROMs) and QoL within 24 months post-surgery*
    a. KOOS (overall and at component level)
    b. Pain Visual Analogue Scale (VAS) (26, 27)
    c. Oxford Knee Score (OKS) (28-30)
2. *Objective assessment of knee function*
    a. Active range of movement

For peer review only

b. Timed-up-and-go test(31, 32)

3. *Incidence of complications, including infection*
    a. Intra-operative complications
    b. Post-operative complications(33)
4. *Further interventions within 24 months post-surgery*
    a. Further surgical interventions including conversion to KR or revision surgery
5. *KJD's potential as cartilage regenerative therapy*
    a. Joint space width (assessed using standardised fixed-flexion PA at 20º X-rays (34))
6. *Estimate of short- and long-term cost-effectiveness*
    a. EQ-5D-3L questionnaire at 24 months
    b. Health Resource Utilisation and Private Costs questionnaire at 24 months
    c. Incremental costs per Quality Adjusted Life Years (QALY) gained at 20 years
7. *Implementation processes and intervention fidelity*
    a. Quantitative *(*surgical CRF and central review of post-operative x-rays).
    b. Qualitative evaluation with surgical and clinical staff
8. *Qualitative evaluation of participant experiences*

Statistical Considerations and Analyses

<u>Sample size</u>

Power calculations are based on a non-inferiority hypothesis for the primary outcome measure, KOOS pain score. 344 participants (172 per arm) will have 90% power to demonstrate non-inferiority based on an 8 point non-inferiority margin, assuming a standard deviation of 21 points (2, 35-37), one-sided 2.5% significance level and 15% dropout rate. The non-inferiority margin was agreed by clinical and patient co-applicants based on being 33% less than the 12 point minimally important difference observed in previous trials (18, 38-40), clinical co-applicant experience and PPI focus group feedback. No adjustment has been made to accommodate surgeon learning curve since external fixation is a common procedure orthopaedic surgeons frequently do for trauma, and minimum expertise is required for surgeon eligibility.

<u>Analysis methods</u>

Full statistical analysis plan predefining all analyses and patient populations will be in place prior to any comparative analyses according to guidelines (41). KARDS will be reported according to the CONSORT extension for Non-Inferiority and Equivalence Randomized Trials (42). The intention-to-treat (ITT) population will include all randomised participants, and the per-protocol (PP) population will include all participants who received their randomised intervention as intended. Although there is no 'gold standard' for non-inferiority trials, outcomes will be analysed primarily for the PP population (43). A sensitivity analysis will be conducted for the ITT population.

For peer review only

The primary analysis will report adjusted estimates of treatment effect from multivariable regression of KOOS pain score at 12 months. Statistical significance of KJD non-inferiority relative to KR will be based on a 2-sided likelihood-based test with type 1 error of 2.5% in both tails, adjusted by baseline score and OA severity as fixed effects, and delivery unit as a random effect (44). If the 95% confidence interval for absolute difference in means between KJD and KR lies entirely below or includes the non-inferiority boundary then there would be insufficient evidence to reject the null hypothesis that KJD inferior to KR. Conversely, if the 95% confidence interval lies entirely above the non-inferiority boundary, there would be evidence to reject the null hypothesis and conclude KJD non-inferior to KR. If non-inferiority demonstrated and KJD appears superior to KR, based on estimated effect and associated confidence interval, statistical significance for superiority will be calculated based on an ITT analysis. Secondary analysis of the primary outcome measure will use multilevel modelling to account for longitudinal data collected over 24 months. Sensitivity analyses will be considered to investigate any impact of surgeon experience on treatment effect estimates(45).

Reasons for missing data will be examined and primary method to account for missing data will be chosen based on the most reasonable missing data mechanism assumption, with sensitivity analyses to assess robustness of results to different missing data mechanism assumptions.

Other PROM responses will be transformed into dimension scores, according to scoring manuals, and presented graphically and longitudinally. Standardised area under the curve (AUC) statistics will be compared across treatment groups as an analysis conditional on patient time in the trial. Functional assessments will be reported descriptively, along with joint space width for the KJD group.

Complications will be reported as unique events and unique patients experiencing events. Joint survival will be measured from randomisation to time of further intervention and analysed using the Kaplan Meier method.

Process evaluation interview data will be analysed using thematic content analysis to identify patterns or themes (46), using coding of audio-transcript recordings, adopting the framework method described by Ritchie and Spencer and Pope et al (47, 48). Normalisation Process Theory will be used as a theoretical framework to explore and explain extent of intervention implementation (49-51), using the software package NVivo 12 to manage data and facilitate this process. Interview data and full record of issues raised will be discussed in detail with the Trial Management Group and summarised for oversight committees. Good practice will be shared with other recruiting sites.

Cost-effectiveness analysis will be conducted from NHS and Personal Social Services perspectives and society over a 24-month time horizon. The analysis will estimate surgical intervention costs and primary and secondary health care services costs including complications, follow-up, medications and repeat medical procedures, and out of pocket and productivity costs to patients and their families. Outcomes will be evaluated using QALYs estimated by the AUC approach. Unit costs will be obtained from list prices for devices and materials involved in the interventions, medications list prices, NHS health professional staff salary scales,

For peer review only

primary care and community services opportunity costs (52), outpatient, inpatient admissions and Accident and Emergency visits NHS Reference Costs , and median UK gross hourly earnings(53). Generalised linear models will be used to adjust for unbalanced baseline covariates in costs (54, 55) and adjusting for baseline EQ-5D-3L score in analysing QALYs (56). Missing data will be imputed using established methods (57). Results will be presented in terms of incremental cost per QALY gained and cost per unit gain in 12-month KOOS. Sampling uncertainty will be analysed using the bootstrap method (58) and joint uncertainty in costs and QALYs will be analysed using cost-effectiveness acceptability curves (59).

A decision analytic model will be built to evaluate lifetime cost-effectiveness over 20 years by adapting and updating a published Markov model of delayed joint replacement using National Joint Registry, clinical study and UK life table data (9, 10). The model will account for trade-offs of delaying knee replacement in terms of reducing the risk of the patient requiring revision surgery near end of life and increased complication risk with primary operation at older age (9). Sampling uncertainty in model parameter values will be described using probabilistic sensitivity analysis, while key parameters affecting the likelihood of KJD meeting the NICE £20,000 threshold for cost-effectiveness (60) will be identified using Tornado plots.

Monitoring

An independent Trial Steering Committee (TSC), comprising a Statistician, two orthopaedic consultant surgeons and one patient representative, will have overall responsibility for trial oversight, monitoring trial progress, protocol adherence and participant safety. An independent Data Monitoring and Ethics Committee (DMEC) comprising a Statistician and two orthopaedic consultant surgeons, will review interim safety data by randomised group, reviewing the underlying statistical design assumptions to ensure the trial remains adequately powered. TSC and DMEC meetings will be conducted annually as a minimum according to agreed TSC and DMEC Charters (61).

No formal guidelines for stopping the trial early are in place since no formal planned interim analysis of the primary outcome is planned.

Information on complications will be collected from randomisation to end of trial defined as the last visit date of the last patient. Serious complications will be subject to expedited reporting where sites will inform CTRU within 24 hours of becoming aware of it. Suspected or confirmed pregnancies and all deaths from randomisation until the end of trial will be reported to CTRU.

Governance, ethics and dissemination

For peer review only

KARDS is funded by NIHR HTA (reference: 17/122/06) and sponsored by the University of Leeds, approved by the Research Ethics Committee (REC) (reference: 19/YH/0368) and Health Research Authority (HRA). All amendments will be submitted for approval and communicated to sites in accordance with HRA guidelines.

Trial results will be disseminated at relevant clinical conferences and societies, published in peer-reviewed journals and disseminated through relevant patient groups. Authorship will be according to ICMJE guidelines.

Discussion

KARDS is a pragmatic, multi-centre prospective randomised controlled trial conducted in an NHS setting, the aim is to determine if KJD is non-inferior to KR in terms of pain and cost-effective in the NHS then it could be routinely offered to patients aged 65 years or less. In addition, it will report on radiological outcomes and patient acceptability. It will be a definitive IDEAL Stage 3 (Assessment) trial (21) with potential to lead to a paradigm shift if it demonstrates non-inferiority of KJD compared to KR.

Joint distraction outcomes at various anatomical locations have been reported in several case series. Though small numbers of patients have been involved, results are encouraging in at least providing temporary symptom relief. At the ankle, improvements in reported symptoms were seen in 73-91% of patients at mean follow up time of 1-12 years (62). Joint distraction has been demonstrated to give good clinical outcomes in first carpometacarpal joint osteoarthritis, albeit in a very limited number of patients. Patients were followed for 1 year with improved functional scores compared to baseline (63). The KJD literature is difficult to assess due to heterogeneity of devices and methods used. A recent review included one cohort study and two small trials all of which came from the same research group including a total of 62 patients (64). These studies all utilised a spring-loaded static distractor. Western Ontario and McMaster Universities Osteoarthritis Index score improvements were significantly greater one-year post KJD than conservatively managed osteoarthritis (17), and not inferior to total KR (19), or high tibial osteotomy (HTO) (11).

Two studies (11, 19) reported KOOS, Intermittent and Constant Osteoarthritis Pain score, EuroQol 5 Dimensions (EQ-5D) and Short Form (SF)-36 with significant improvements at one year seen in all scores except for the SF-36 mental component score, with no significant difference in these improvements compared to KR or HTO. Pain score assessed on pain VAS was reported in both studies and showed improvements at one year with no significant difference between KJD and HTO or KR. Radiographic assessment of joint structure has been undertaken in various studies, with imaging at the time of distraction or follow up. The group above utilised MRI to assess structural recovery. Mean cartilage thickness was shown to increase on both the tibial and femoral sides and percentage of joint surface appearing as denuded subchondral bone decreased (64). Radiographic minimum joint space width was shown to increase by 0.8mm at 12 months compared to baseline (11, 17, 19), Similar to another study where the mean joint space width, measured using standardised digital techniques, increased from 2.7mm to 3.6mm 12 months post-fixator removal (65).

For peer review only

The most frequently reported KJD complication is pin site infection. Rates approaching 70% have been reported, with 20% of affected patients requiring intravenous therapy (64). In the series of 62 patients described above, two patients required surgical intervention for pin-site infection during distraction, with a further case of osteomyelitis requiring surgery following fixator removal (11, 17, 19). These infection rates are at odds with those reported in patients treated by definitive external fixation for other reasons. Pin-site infection rates of 40% are found fairly consistently, even where fixators are in place for much longer, the reasons for this are unclear (66). Whilst transient pin-site infection seldom has long term implications, it is unpleasant for patients and may impair rehabilitation. Deep infections may be more worrisome, especially considering expected osteoarthritis progression following distraction potentially requiring eventual arthroplasty. Wherever possible, external fixator pins will be sited outside the implantation zone of a KR. Total KR following significant osteomyelitis is significantly more complex and has further infection risk even when infection considered eradicated (67). Current KJD literature does not provide sufficient evidence to estimate serious infection rates following conversion to KR. In one ankle distraction study with over five years follow up, there was no infection seen in five patients who had conversion to arthroplasty (68). Loss of knee range of movement immediately following distraction therapy has been observed to return after 1 year, with a small number of patients undergoing joint manipulation under anaesthetic to achieve this (13, 19).

Trial strengths include its pragmatic nature, standardised surgical and assessment techniques, robust reporting and safety mechanisms and appropriate sample size. The window of six weeks between baseline measures and planned surgery date aligns with clinical pathways and ensures recruitment feasibility. KARDS has a pragmatic hybrid expertise-based design, where surgeons are categorised into “delivery units” based on their experience, a successful approach successful for similar knee OA surgical trials (69). Furthermore, clinicians are free to choose KR implant type and KJD external fixator. This choice brings a limitation in not being able to determine potential individual mechanisms of action limiting individual indications and/or contraindications. Those implants and fixators approved in the trial protocol are based upon consensus amongst experts and published literature. Sub-group analysis will not be adequately powered to determine if a particular fixator type is superior. A further limitation is the lack of blinding but this is unavoidable. It would be impractical to blind medical staff prior to surgery at many sites as they need to plan for the specific surgery. PPI feedback was that being blinded until just before or after surgery would be unacceptable if the medical team knew the allocation. The primary outcome measure is patient reported and therefore it is not possible to have a blinded primary outcome assessment.

Declaration of interests

HKS – Consultant to Orthofix and received research grant from Orthofix. Received payment for teaching responsibilities from Orthofix and Smith & Nephew.

For peer review only

HP - received grant funding from KTP, Pacira Pharmaceuticals, Zimmer Biomet Healthcare, B Braun & Welcome Trust. Received consulting fees from Medacta International, Smith and Nephew, Depuy Synthes, JRI Orthopaedics, Janssen, Meril Life, Zimmer Biomet & Paradigm Pharmaceuticals. Received payment from Invibio for presentations, from Kennedy's Law for expert testimony & from Pacira Pharmaceuticals for study conduct. Received payments from Medacta International, Depuy Synthes & Zimmer Biomet for attending meetings/travel.

AHRWS - received grant funding from EPSRC Ultrasonic Surgery & EPSRC 2050 EnLightenus. Submitted patent with Joint Assist patient application.

AM – received grant funding for from Stryker for the RACER-Hip trial.

Authors' contributions

KARDS study design: RH, IS, DDS, HP, CT, AM, AHRWS, DM, DMc, HKS, TWH, DE, CF, RL, JC, PJH. Writing of manuscript: RH, CT, IS, DDS, HP, SA, PJH, RMM, AHRWS. All authors have read and approved of the final manuscript.

Funding

This study is funded by the National Institute for Health Research (NIHR) Health Technology Assessment (HTA) Programme (Project: 17/122/06). The views expressed are those of the authors and not necessarily those of the NIHR or Department of Health and Social Care.

Access to data

To maintain scientific integrity of the trial, outcome data will not be released prior to publication of the analysis of the primary outcome measure, either for trial publication or oral presentation purposes, without permission of the TSC. Following publication, requests for sharing of trial data may be submitted to Leeds CTRU.

References

1. Lawrence RC, Felson DT, Helmick CG, Arnold LM, Choi H, Deyo RA, et al. Estimates of the prevalence of arthritis and other rheumatic conditions in the United States. Part II. Arthritis and rheumatism. 2008;58(1):26-35.
2. Skou ST, Roos EM, Laursen MB, Rathleff MS, Arendt-Nielsen L, Simonsen O, et al. A Randomized, Controlled Trial of Total Knee Replacement. The New England journal of medicine. 2015;373(17):1597-606.
3. Bayliss LE, Culliford D, Monk AP, Glyn-Jones S, Prieto-Alhambra D, Judge A, et al. The effect of patient age at intervention on risk of implant revision after total replacement of the hip or knee: a population-based cohort study. Lancet (London, England). 2017;389(10077):1424-30.
4. Deehan DJ, Murray JD, Birdsall PD, Pinder IM. Quality of life after knee revision arthroplasty. Acta orthopaedica. 2006;77(5):761-6.
5. Stambough JB, Clohisy JC, Barrack RL, Nunley RM, Keeney JA. Increased risk of failure following revision total knee replacement in patients aged 55 years and younger. The bone & joint journal. 2014;96-b(12):1657-62.

For peer review only

6. About the James Lind Alliance: James Lind Alliance [Internet]; [cited 2018 Jul 25] [Available from: http://www.jla.nihr.ac.uk/about-the-james-lind-alliance/.
7. Price AJ, Longino D, Rees J, Rout R, Pandit H, Javaid K, et al. Are pain and function better measures of outcome than revision rates after TKR in the younger patient? The Knee. 2010;17(3):196-9.
8. Bitton R. The economic burden of osteoarthritis. The American journal of managed care. 2009;15(8 Suppl):S230-5.
9. Mota RE. Cost-effectiveness analysis of early versus late total hip replacement in Italy. Value Health. 2013;16(2):267-79.
10. Lan RH, Yu J, Samuel LT, Pappas MA, Brooks PJ, Kamath AFJTJoA. How are we measuring cost-effectiveness in total joint arthroplasty studies? Systematic review of the literature. 2020;35(11):3364-74.
11. van der Woude JAD, Wiegant K, van Heerwaarden RJ, Spruijt S, van Roermund PM, Custers RJH, et al. Knee joint distraction compared with high tibial osteotomy: a randomized controlled trial. Knee Surg Sports Traumatol Arthrosc. 2017;25(3):876-86.
12. Jansen MP, Besselink NJ, van Heerwaarden RJ, Custers RJH, Emans PJ, Spruijt S, et al. Knee Joint Distraction Compared with High Tibial Osteotomy and Total Knee Arthroplasty: Two-Year Clinical, Radiographic, and Biochemical Marker Outcomes of Two Randomized Controlled Trials. Cartilage. 2021;12(2):181-91.
13. Jansen MP, Mastbergen SC, van Heerwaarden RJ, Spruijt S, van Empelen MD, Kester EC, et al. Knee joint distraction in regular care for treatment of knee osteoarthritis: A comparison with clinical trial data. PLoS One. 2020;15(1):e0227975.
14. Wiegant K, van Roermund PM, Intema F, Cotofana S, Eckstein F, Mastbergen SC, et al. Sustained clinical and structural benefit after joint distraction in the treatment of severe knee osteoarthritis. Osteoarthritis and cartilage. 2013;21(11):1660-7.
15. Bellamy N, Buchanan WW, Goldsmith CH, Campbell J, Stitt LW. Validation study of WOMAC: a health status instrument for measuring clinically important patient relevant outcomes to antirheumatic drug therapy in patients with osteoarthritis of the hip or knee. The Journal of rheumatology. 1988;15(12):1833-40.
16. Lequesne M. Indices of severity and disease activity for osteoarthritis. Seminars in arthritis and rheumatism. 1991;20(6 Suppl 2):48-54.
17. van der Woude JAD, Wiegant K, van Roermund PM, Intema F, Custers RJH, Eckstein F, et al. Five-Year Follow-up of Knee Joint Distraction: Clinical Benefit and Cartilaginous Tissue Repair in an Open Uncontrolled Prospective Study. Cartilage. 2017;8(3):263-71.
18. van der Woude JAD, Wiegant K, van Heerwaarden RJ, Spruijt S, Emans PJ, Mastbergen SC, et al. Knee joint distraction compared with total knee arthroplasty. 2017;99-B(1):51-8.
19. van der Woude JAD, Nair SC, Custers RJH, van Laar JM, Kuchuck NO, Lafeber FP, et al. Knee Joint Distraction Compared to Total Knee Arthroplasty for Treatment of End Stage Osteoarthritis: Simulating Long-Term Outcomes and Cost-Effectiveness. PLoS One. 2016;11(5):e0155524.
20. Wiegant K, Intema F, van Roermund PM, Barten-van Rijbroek AD, Doornebal A, Hazewinkel HA, et al. Evidence of cartilage repair by joint distraction in a canine model of osteoarthritis. Arthritis & rheumatology (Hoboken, NJ). 2015;67(2):465-74.
21. Hirst A, Philippou Y, Blazeby J, Campbell B, Campbell M, Feinberg J, et al. No Surgical Innovation Without Evaluation: Evolution and Further Development of the IDEAL Framework and Recommendations. 2019;269(2):211-20.
22. Blencowe N, Mills N, Cook J, Donovan J, Rogers C, Whiting P, et al. Standardizing and monitoring the delivery of surgical interventions in randomized clinical trials. 2016;103(10):1377-84.
23. Kellgren JH, Lawrence JS. Radiological assessment of osteo-arthrosis. Annals of the rheumatic diseases. 1957;16(4):494-502.

For peer review only

24. Chan A-W, Tetzlaff JM, Gøtzsche PC, Altman DG, Mann H, Berlin JA, et al. SPIRIT 2013 explanation and elaboration: guidance for protocols of clinical trials. BMJ : British Medical Journal. 2013;346(jan08 15):e7586-e.
25. Rolfson O, Wissig S, van Maasakkers L, Stowell C, Ackerman I, Ayers D, et al. Defining an International Standard Set of Outcome Measures for Patients With Hip or Knee Osteoarthritis: Consensus of the International Consortium for Health Outcomes Measurement Hip and Knee Osteoarthritis Working Group. Arthritis care & research. 2016;68(11):1631-9.
26. Hawker GA, Mian S, Kendzerska T, French M. Measures of adult pain: Visual Analog Scale for Pain (VAS Pain), Numeric Rating Scale for Pain (NRS Pain), McGill Pain Questionnaire (MPQ), Short-Form McGill Pain Questionnaire (SF-MPQ), Chronic Pain Grade Scale (CPGS), Short Form-36 Bodily Pain Scale (SF-36 BPS), and Measure of Intermittent and Constant Osteoarthritis Pain (ICOAP). Arthritis care & research. 2011;63 Suppl 11:S240-52.
27. McCormack HM, Horne DJ, Sheather S. Clinical applications of visual analogue scales: a critical review. Psychological medicine. 1988;18(4):1007-19.
28. Dawson J, Fitzpatrick R, Murray D, Carr A. Questionnaire on the perceptions of patients about total knee replacement. The Journal of bone and joint surgery British volume. 1998;80(1):63-9.
29. Dunbar MJ, Robertsson O, Ryd L, Lidgren L. Appropriate questionnaires for knee arthroplasty. Results of a survey of 3600 patients from The Swedish Knee Arthroplasty Registry. The Journal of bone and joint surgery British volume. 2001;83(3):339-44.
30. Xie F, Ye H, Zhang Y, Liu X, Lei T, Li SC. Extension from inpatients to outpatients: validity and reliability of the Oxford Knee Score in measuring health outcomes in patients with knee osteoarthritis. International journal of rheumatic diseases. 2011;14(2):206-10.
31. CDC. Timed Up & Go [Available from: https://www.cdc.gov/steadi/pdf/TUG_test-print.pdf.
32. Davis AM, King LK, Stanaitis I, Hawker GAJO, Cartilage. Fundamentals of osteoarthritis: outcome evaluation with patient-reported measures and functional tests. 2021.
33. GOV.UK. Surgical site infection surveillance service: protocol and guides [Internet] [cited 2019 Sep 9] [Available from: https://www.gov.uk/government/publications/surgical-site-infection-surveillance-service-protocol-procedure-codes-and-user-manual.
34. Marijnissen AC, Vincken KL, Vos PA, Saris DB, Viergever MA, Bijlsma JW, et al. Knee Images Digital Analysis (KIDA): a novel method to quantify individual radiographic features of knee osteoarthritis in detail. Osteoarthritis and cartilage. 2008;16(2):234-43.
35. A WD, Toksvig-Larsen S, Roos EM. Association between knee alignment and knee pain in patients surgically treated for medial knee osteoarthritis by high tibial osteotomy. A one year follow-up study. BMC musculoskeletal disorders. 2009;10:154.
36. Nilsdotter AK, Toksvig-Larsen S, Roos EM. Knee arthroplasty: are patients' expectations fulfilled? A prospective study of pain and function in 102 patients with 5-year follow-up. Acta orthopaedica. 2009;80(1):55-61.
37. Roos EM, Toksvig-Larsen S. Knee injury and Osteoarthritis Outcome Score (KOOS) - validation and comparison to the WOMAC in total knee replacement. Health and quality of life outcomes. 2003;1:17.
38. State of Musculoskeletal Health 2017: Arthritis Research UK; 2017 [
39. Collins NJ, Misra D, Felson DT, Crossley KM, Roos EM. Measures of knee function: International Knee Documentation Committee (IKDC) Subjective Knee Evaluation Form, Knee Injury and Osteoarthritis Outcome Score (KOOS), Knee Injury and Osteoarthritis Outcome Score Physical Function Short Form (KOOS-PS), Knee Outcome Survey Activities of Daily Living Scale (KOS-ADL), Lysholm Knee Scoring Scale, Oxford Knee Score (OKS), Western Ontario and McMaster Universities Osteoarthritis Index (WOMAC), Activity Rating Scale (ARS), and Tegner Activity Score (TAS). Arthritis care & research. 2011;63 Suppl 11(0 11):S208-28.
40. Devji T, Guyatt GH, Lytvyn L, Brignardello-Petersen R, Foroutan F, Sadeghirad B, et al. Application of minimal important differences in degenerative knee disease outcomes: a

For peer review only

systematic review and case study to inform BMJ Rapid Recommendations. 2017;7(5):e015587.
41. Gamble C, Krishan A, Stocken D, Lewis S, Juszczak E, Doré C, et al. Guidelines for the Content of Statistical Analysis Plans in Clinical Trials. Jama. 2017;318(23):2337-43.
42. Piaggio G, Elbourne DR, Pocock SJ, Evans SJ, Altman DG. Reporting of noninferiority and equivalence randomized trials: extension of the CONSORT 2010 statement. Jama. 2012;308(24):2594-604.
43. Rehal S, Morris TP, Fielding K, Carpenter JR, Phillips PP. Non-inferiority trials: are they inferior? A systematic review of reporting in major medical journals. BMJ open. 2016;6(10):e012594.
44. Beard DJ, Davies LJ, Cook JA, MacLennan G, Price A, Kent S, et al. The clinical and cost-effectiveness of total versus partial knee replacement in patients with medial compartment osteoarthritis (TOPKAT): 5-year outcomes of a randomised controlled trial. The Lancet. 2019;394(10200):746-56.
45. Corrigan N, Marshall H, Croft J, Copeland J, Jayne D, Brown J. Exploring and adjusting for potential learning effects in ROLARR: a randomised controlled trial comparing robotic-assisted vs. standard laparoscopic surgery for rectal cancer resection. Trials. 2018;19(1):339.
46. Braun V, Clarke V. Using thematic analysis in psychology. Qualitative Research in Psychology. 2006;3(2):77-101.
47. NICE. Guide to the methods of technology appraisal 2013. NICE; 2013.
48. Pope C, Mays N. Reaching the parts other methods cannot reach: an introduction to qualitative methods in health and health services research. BMJ (Clinical research ed). 1995;311(6996):42-5.
49. May C, Finch T. Implementing, Embedding, and Integrating Practices: An Outline of Normalization Process Theory. 2009;43(3):535-54.
50. May C, Finch T, Mair F, Ballini L, Dowrick C, Eccles M, et al. Understanding the implementation of complex interventions in health care: the normalization process model. BMC Health Services Research. 2007;7(1):148.
51. May CR, Mair F, Finch T, MacFarlane A, Dowrick C, Treweek S, et al. Development of a theory of implementation and integration: Normalization Process Theory. Implementation Science. 2009;4(1):29.
52. Curtis L, Burns A. Unit Costs of Health and Social Care 2020, Personal Social Services Research Unit, University of Kent, Canterbury. 2020.
53. Statistics OfN. Annual Survey of Hours and Earnings (ASHE) 2020. [Available from: https://www.ons.gov.uk/employmentandlabourmarket/peopleinwork/earningsandworkinghours/
54. Mihaylova B, Briggs A, O'Hagan A, Thompson SG. Review of statistical methods for analysing healthcare resources and costs. Health economics. 2011;20(8):897-916.
55. Thompson SG, Nixon RM. How sensitive are cost-effectiveness analyses to choice of parametric distributions? Medical decision making : an international journal of the Society for Medical Decision Making. 2005;25(4):416-23.
56. Manca A, Hawkins N, Sculpher MJ. Estimating mean QALYs in trial-based cost-effectiveness analysis: the importance of controlling for baseline utility. Health economics. 2005;14(5):487-96.
57. Faria R, Gomes M, Epstein D, White IR. A guide to handling missing data in cost-effectiveness analysis conducted within randomised controlled trials. PharmacoEconomics. 2014;32(12):1157-70.
58. Briggs AH, Wonderling DE, Mooney CZ. Pulling cost-effectiveness analysis up by its bootstraps: a non-parametric approach to confidence interval estimation. Health economics. 1997;6(4):327-40.
59. Fenwick E, Claxton K, Sculpher M. Representing uncertainty: the role of cost-effectiveness acceptability curves. Health economics. 2001;10(8):779-87.

For peer review only - http://bmjopen.bmj.com/site/about/guidelines.xhtml

60. National Institute for H, Care E. NICE Process and Methods Guides. Guide to the Methods of Technology Appraisal 2013. London: National Institute for Health and Care Excellence (NICE)

Copyright © 2013 National Institute for Health and Clinical Excellence, unless otherwise stated. All rights reserved.; 2013.
61. A proposed charter for clinical trial data monitoring committees: helping them to do their job well. Lancet (London, England). 2005;365(9460):711-22.
62. van Valburg AA, van Roermund PM, Lammens J, van Melkebeek J, Verbout AJ, Lafeber EP, et al. Can Ilizarov joint distraction delay the need for an arthrodesis of the ankle? A preliminary report. The Journal of bone and joint surgery British volume. 1995;77(5):720-5.
63. Spaans AJ, Minnen LPV, Braakenburg A, Mink van der Molen AB. Joint distraction for thumb carpometacarpal osteoarthritis: a feasibility study with 1-year follow-up. Journal of plastic surgery and hand surgery. 2017;51(4):254-8.
64. Takahashi T, Baboolal TG, Lamb J, Hamilton TW, Pandit HG. Is Knee Joint Distraction a Viable Treatment Option for Knee OA?-A Literature Review and Meta-Analysis. The journal of knee surgery. 2019;32(8):788-95.
65. Intema F, Van Roermund PM, Marijnissen AC, Cotofana S, Eckstein F, Castelein RM, et al. Tissue structure modification in knee osteoarthritis by use of joint distraction: an open 1-year pilot study. Annals of the rheumatic diseases. 2011;70(8):1441-6.
66. Ferguson D, Harwood P, Allgar V, Roy A, Foster P, Taylor M, et al. The PINS Trial: a prospective randomized clinical trial comparing a traditional versus an emollient skincare regimen for the care of pin-sites in patients with circular frames. The bone & joint journal. 2021;103-b(2):279-85.
67. Williams RL, Khan W, Roberts-Huntleigh N, Morgan-Jones R. Total knee arthroplasty in patients with prior adjacent multi-organism osteomyelitis. Acta orthopaedica Belgica. 2018;84(2):184-91.
68. Nguyen MP, Pedersen DR, Gao Y, Saltzman CL, Amendola A. Intermediate-term follow-up after ankle distraction for treatment of end-stage osteoarthritis. The Journal of bone and joint surgery American volume. 2015;97(7):590-6.
69. Beard D, Price A, Cook J, Fitzpatrick R, Carr A, Campbell M, et al. Total or Partial Knee Arthroplasty Trial - TOPKAT: study protocol for a randomised controlled trial. Trials. 2013;14(1):292.

For peer review only

	Screening and Consent	Randomisation & Baseline		Surgery (Day 0)	Follow-up time point						Unscheduled
					Clinic visits			Postal questionnaire packs	Clinic visits		
	Prior to registration	Before randomisation and within 6 weeks prior to surgery	After randomisation and within 6 weeks prior to surgery		Post-operative (Up to Day 7)	Fixator Removal (Week 6)	Follow up (Month 3)	Follow up (Month 6)	Follow up (Month 12)	Follow up (Month 24)	
	All	All	All	All	All	KJD arm only	All	All	All	All	All
Informed Consent	X										
Screening Data	X										
Eligibility	X										
Patient Details	X										
Patient Demographics		X									
Medical History		X									
OA Severity (Kellgren-Lawrence grade based on standard AP & lateral x-rays)		X									
Physical examination of knee		X									
TUG (Timed up and go test)		X					X		X	X	
ROM (Range of movement) using goniometer		X					X		X	X	
Rosenberg View X-ray			X				X^		X^	X^	
Surgery (KR or KJD)				X							
Surgical details				X		X^					
Distraction of external fixator (KJD only)				X^	X^						
Removal of external fixator (KJD only)						X^					
Intra-operative Complications				X		X^					
Additional knee related and/or other limb surgery				X	X	X^	X	X	X	X	X
Concomitant Medications				X	X	X^	X	X	X	X	X
Discharge Details					X	X^					

For peer review only

AP/Lateral View X-rays					X	X^						
Post-operative Complications					X	X^	X	X	X	X	X	
**Patient Reported Outcomes**												
KOOS		X		X*			X	X	X	X		
OKS		X					X	X	X	X		
EQ5D-3L		X					X	X	X	X		
Pain VAS		X					X	X	X	X		
Health Resource Use		X					X	X	X	X		
Serious complications											X	
Participant withdrawal											X	
Re-operation											X	
Pregnancy											X	
Death											X	

*Up to 1 day before surgery
^KJD arm only

For peer review only

SPIRIT 2013 Checklist: Recommended items to address in a clinical trial protocol and related documents*

Section/item	Item No	Description	Addressed on page number
**Administrative information**			
Title	1	Descriptive title identifying the study design, population, interventions, and, if applicable, trial acronym	1
Trial registration	2a	Trial identifier and registry name. If not yet registered, name of intended registry	2
	2b	All items from the World Health Organization Trial Registration Data Set	11
Protocol version	3	Date and version identifier	4
Funding	4	Sources and types of financial, material, and other support	11
Roles and responsibilities	5a	Names, affiliations, and roles of protocol contributors	1
	5b	Name and contact information for the trial sponsor	11
	5c	Role of study sponsor and funders, if any, in study design; collection, management, analysis, and interpretation of data; writing of the report; and the decision to submit the report for publication, including whether they will have ultimate authority over any of these activities	11-12
	5d	Composition, roles, and responsibilities of the coordinating centre, steering committee, endpoint adjudication committee, data management team, and other individuals or groups overseeing the trial, if applicable (see Item 21a for data monitoring committee)	11

For peer review only

For peer review only - http://bmjopen.bmj.com/site/about/guidelines.xhtml

Introduction

Background and rationale	6a	Description of research question and justification for undertaking the trial, including summary of relevant studies (published and unpublished) examining benefits and harms for each intervention	_____3-4_____
	6b	Explanation for choice of comparators	______3______
Objectives	7	Specific objectives or hypotheses	______4______
Trial design	8	Description of trial design including type of trial (eg, parallel group, crossover, factorial, single group), allocation ratio, and framework (eg, superiority, equivalence, noninferiority, exploratory)	______4______

Methods: Participants, interventions, and outcomes

Study setting	9	Description of study settings (eg, community clinic, academic hospital) and list of countries where data will be collected. Reference to where list of study sites can be obtained	_____4-5_____
Eligibility criteria	10	Inclusion and exclusion criteria for participants. If applicable, eligibility criteria for study centres and individuals who will perform the interventions (eg, surgeons, psychotherapists)	______5-6_____
Interventions	11a	Interventions for each group with sufficient detail to allow replication, including how and when they will be administered	______6-7_____
	11b	Criteria for discontinuing or modifying allocated interventions for a given trial participant (eg, drug dose change in response to harms, participant request, or improving/worsening disease)	______NA_____
	11c	Strategies to improve adherence to intervention protocols, and any procedures for monitoring adherence (eg, drug tablet return, laboratory tests)	6; surgical manual 5; hybrid-expertise design
	11d	Relevant concomitant care and interventions that are permitted or prohibited during the trial	______7______
Outcomes	12	Primary, secondary, and other outcomes, including the specific measurement variable (eg, systolic blood pressure), analysis metric (eg, change from baseline, final value, time to event), method of aggregation (eg, median, proportion), and time point for each outcome. Explanation of the clinical relevance of chosen efficacy and harm outcomes is strongly recommended	_____8-9_____

For peer review only - http://bmjopen.bmj.com/site/about/guidelines.xhtml

Participant timeline	13	Time schedule of enrolment, interventions (including any run-ins and washouts), assessments, and visits for participants. A schematic diagram is highly recommended (see Figure)	______8______
Sample size	14	Estimated number of participants needed to achieve study objectives and how it was determined, including clinical and statistical assumptions supporting any sample size calculations	______9_____
Recruitment	15	Strategies for achieving adequate participant enrolment to reach target sample size	4 & 8; pilot phase & qualitative process

Methods: Assignment of interventions (for controlled trials)

Allocation:

Sequence generation	16a	Method of generating the allocation sequence (eg, computer-generated random numbers), and list of any factors for stratification. To reduce predictability of a random sequence, details of any planned restriction (eg, blocking) should be provided in a separate document that is unavailable to those who enrol participants or assign interventions	______7______
Allocation concealment mechanism	16b	Mechanism of implementing the allocation sequence (eg, central telephone; sequentially numbered, opaque, sealed envelopes), describing any steps to conceal the sequence until interventions are assigned	______7______
Implementation	16c	Who will generate the allocation sequence, who will enrol participants, and who will assign participants to interventions	______7______
Blinding (masking)	17a	Who will be blinded after assignment to interventions (eg, trial participants, care providers, outcome assessors, data analysts), and how	______7______
	17b	If blinded, circumstances under which unblinding is permissible, and procedure for revealing a participant's allocated intervention during the trial	______NA______

Methods: Data collection, management, and analysis

Data collection methods	18a	Plans for assessment and collection of outcome, baseline, and other trial data, including any related processes to promote data quality (eg, duplicate measurements, training of assessors) and a description of study instruments (eg, questionnaires, laboratory tests) along with their reliability and validity, if known. Reference to where data collection forms can be found, if not in the protocol	_____8-9______
	18b	Plans to promote participant retention and complete follow-up, including list of any outcome data to be collected for participants who discontinue or deviate from intervention protocols	______8______
Data management	19	Plans for data entry, coding, security, and storage, including any related processes to promote data quality (eg, double data entry; range checks for data values). Reference to where details of data management procedures can be found, if not in the protocol	______8______
Statistical methods	20a	Statistical methods for analysing primary and secondary outcomes. Reference to where other details of the statistical analysis plan can be found, if not in the protocol	_____9-11_____
	20b	Methods for any additional analyses (eg, subgroup and adjusted analyses)	_____9-10_____
	20c	Definition of analysis population relating to protocol non-adherence (eg, as randomised analysis), and any statistical methods to handle missing data (eg, multiple imputation)	______9______
**Methods: Monitoring**			
Data monitoring	21a	Composition of data monitoring committee (DMC); summary of its role and reporting structure; statement of whether it is independent from the sponsor and competing interests; and reference to where further details about its charter can be found, if not in the protocol. Alternatively, an explanation of why a DMC is not needed	______11_____
	21b	Description of any interim analyses and stopping guidelines, including who will have access to these interim results and make the final decision to terminate the trial	______11_____
Harms	22	Plans for collecting, assessing, reporting, and managing solicited and spontaneously reported adverse events and other unintended effects of trial interventions or trial conduct	______11_____
Auditing	23	Frequency and procedures for auditing trial conduct, if any, and whether the process will be independent from investigators and the sponsor	______11_____
**Ethics and dissemination**			

Research ethics approval	24	Plans for seeking research ethics committee/institutional review board (REC/IRB) approval	_______11_____
Protocol amendments	25	Plans for communicating important protocol modifications (eg, changes to eligibility criteria, outcomes, analyses) to relevant parties (eg, investigators, REC/IRBs, trial participants, trial registries, journals, regulators)	_______12_____
Consent or assent	26a	Who will obtain informed consent or assent from potential trial participants or authorised surrogates, and how (see Item 32)	_______5______
	26b	Additional consent provisions for collection and use of participant data and biological specimens in ancillary studies, if applicable	_______NA_____
Confidentiality	27	How personal information about potential and enrolled participants will be collected, shared, and maintained in order to protect confidentiality before, during, and after the trial	_______5______
Declaration of interests	28	Financial and other competing interests for principal investigators for the overall trial and each study site	_____13-14_____
Access to data	29	Statement of who will have access to the final trial dataset, and disclosure of contractual agreements that limit such access for investigators	_______14_____
Ancillary and post-trial care	30	Provisions, if any, for ancillary and post-trial care, and for compensation to those who suffer harm from trial participation	_______NA_____
Dissemination policy	31a	Plans for investigators and sponsor to communicate trial results to participants, healthcare professionals, the public, and other relevant groups (eg, via publication, reporting in results databases, or other data sharing arrangements), including any publication restrictions	_______13______
	31b	Authorship eligibility guidelines and any intended use of professional writers	_______14______
	31c	Plans, if any, for granting public access to the full protocol, participant-level dataset, and statistical code	_______14______
**Appendices**			
Informed consent materials	32	Model consent form and other related documentation given to participants and authorised surrogates	________NA_____

For peer review only - http://bmjopen.bmj.com/site/about/guidelines.xhtml

Biological specimens	33	Plans for collection, laboratory evaluation, and storage of biological specimens for genetic or molecular analysis in the current trial and for future use in ancillary studies, if applicable	________NA______

*It is strongly recommended that this checklist be read in conjunction with the SPIRIT 2013 Explanation & Elaboration for important clarification on the items. Amendments to the protocol should be tracked and dated. The SPIRIT checklist is copyrighted by the SPIRIT Group under the Creative Commons "Attribution-NonCommercial-NoDerivs 3.0 Unported" license.

For peer review only

Clinical and Cost-effectiveness of Knee Arthroplasty versus Joint Distraction for Osteoarthritis (KARDS): Protocol for A Multicentre, Phase III, Randomised Control Trial

Journal:	*BMJ Open*
Manuscript ID	bmjopen-2022-062721.R1
Article Type:	Protocol
Date Submitted by the Author:	10-May-2022
Complete List of Authors:	Tassinari, Cerys; University of Leeds Leeds Institute of Clinical Trials Research, Statistics<br>Higham, Ruchi; University of Leeds Leeds Institute of Clinical Trials Research, Trial Management<br>Smith, Isabelle; University of Leeds Clinical Trials Research Unit,<br>Arnold, Susanne; University of Warwick, ; The University of Warwick<br>Mujica-Mota, Ruben; University of Leeds, Leeds Institute of Health Sciences<br>Metcalfe, Andrew; University of Warwick Warwick Medical School, Warwick Clinical Trials Unit; University Hospitals Coventry and Warwickshire NHS Trust, Trauma and Orthopaedics<br>Simpson, Hamish; University of Edinburgh,<br>Murray, David; University of Oxford Nuffield Department of Orthopaedics Rheumatology and Musculoskeletal Sciences<br>McGonagle, Dennis; 1. Leeds Institute of Rheumatic and Musculoskeletal Medicine, University of Leeds, NIHR Leeds Biomedical Research Centre, Chapel Allerton Hospital<br>Sharma, Hemant; Hull and East Yorkshire Hospitals NHS Trust, Department of Orthopaedics<br>Hamilton, Thomas; University of Oxford, NDORMS<br>Ellard, David; University of Warwick, Clinical Trials Unit;<br>Fernandez, Catherine; University of Leeds Faculty of Medicine and Health, Clinical Trials Research Unit<br>Reynolds, Catherine; University of Leeds, Clinical Trials Research Unit<br>Harwood, Paul; University of Leeds Leeds Institute of Medical Research<br>Croft, Julie; University of Leeds, Clinical Trials Research Unit, Leeds Institute of Clinical Trials<br>Stocken, Deborah; University of Leeds Leeds Institute of Clinical Trials Research, Statistics<br>Pandit, Hemant ; Chapel Allerton Hospital; 1. Leeds Institute of Rheumatic and Musculoskeletal Medicine, University of Leeds, NIHR Leeds Biomedical Research Centre, Chapel Allerton Hospital
**Primary Subject Heading**:	Surgery
Secondary Subject Heading:	Health economics, Qualitative research
Keywords:	Knee < ORTHOPAEDIC & TRAUMA SURGERY, Musculoskeletal disorders < ORTHOPAEDIC & TRAUMA SURGERY, Orthopaedic & trauma surgery <

	SURGERY, STATISTICS & RESEARCH METHODS

For peer review only - http://bmjopen.bmj.com/site/about/guidelines.xhtml

Clinical and Cost-effectiveness of Knee Arthroplasty versus Joint Distraction for Osteoarthritis (KARDS): Protocol for A Multicentre, Phase III, Randomised Control Trial

Authors and affiliations

Cerys Tassinari[1*], Ruchi Higham[1*], Isabelle Smith[1], Susanne Arnold[2], Ruben Mujica-Mota[3], Andrew Metcalfe[2], A Hamish RW Simpson[4], David Murray[5], Dennis McGonagle[6], Hemant K Sharma[7], Thomas W Hamilton[5], David R Ellard[2], Catherine Fernandez[1], Catherine Reynolds [1], Paul Harwood[8], Julie Croft[1], Deborah D Stocken[1^], Hemant Pandit[6^]

Acknowledgements

Roberta Longo[3]

---

* Joint First Authors

^ Joint Senior Authors

[1] Clinical Trials Research Unit, University of Leeds, UK

[2] Warwick Clinical Trials Unit, University of Warwick, UK

[3] Leeds Institute of Health Sciences, University of Leeds, UK

[4] Department of Orthopaedics and Trauma, University of Edinburgh, UK

[5] Nuffield Department of Orthopaedics Rheumatology and Musculoskeletal Science, University of Oxford, UK

[6] Leeds Institute of Rheumatic and Musculoskeletal Medicine, University of Leeds, UK

[7] Department of Trauma and Orthopaedics, Hull University Teaching Hospitals, Hull, UK

[8] Leeds Institute of Medical Research, University of Leeds, UK

Corresponding author

Cerys Tassinari; C.Tassinari@leeds.ac.uk

Word Count: (excluding title, abstract, references, tables & figures) 4102(Limit: 4000)

Abstract Word Count: 300 (Limit: 300)

For peer review only

ABSTRACT

Introduction

Knee Replacement (KR) is a clinically-proven procedure typically offered to patients with severe knee Osteoarthritis (OA) to relieve pain and improve quality of life. However, artificial joints fail over time, requiring revision associated with higher mortality and inferior outcomes. With more young people presenting with knee OA and increasing life expectancy, there is an unmet need to postpone time to first KR. Knee Joint Distraction (KJD), the practice of using external fixators to open up knee joint space, is proposed as potentially effective to preserve the joint following initial studies in the Netherlands, however, has not been researched within an NHS setting. The KARDS trial will investigate whether KJD is non-inferior to KR in terms of patient-reported post-operative pain 12 months post-surgery.

Methods and analysis

KARDS is a phase III, multi-centre, pragmatic, open-label, individually randomised controlled non-inferiority trial comparing KJD with KR in patients with severe knee OA, employing a hybrid-expertise design, with internal pilot phase and process evaluation. 344 participants will be randomised (1:1) to KJD or KR. The primary outcome measure is the Knee Injury and Osteoarthritis Outcomes Score (KOOS) pain domain score at 12-months post-operation. Secondary outcome measures include patient reported overall KOOS, Pain VAS and Oxford Knee Scores, knee function assessments, joint space width, complications and further interventions over 24 months post-operation. Per patient cost difference between KR and KJD and cost per QALY gained over 24 months will be estimated within trial, and incremental cost per QALY gained over 20 years by KJD relative to KR predicted using decision analytic modelling.

Ethics and dissemination

Ethics approval was obtained from the Research Ethics Committee (REC) and Health Research Authority (HRA). Trial results will be disseminated at clinical conferences, through relevant patient groups and published in peer-reviewed journals.

Trial registration number: ISRCTN14879004. Recruitment opened April 2021.

For peer review only

Strengths and limitations of this trial

- The KARDS trial is a pragmatic trial design using standardised surgical and assessment techniques, robust reporting and safety mechanisms and appropriate sample size.

- A hybrid-expertise based design has been adopted to ensure feasibility of the trial whilst accounting for surgeon experience and potential lack of individual equipoise.

- A surgical manual will document each trial procedure highlighting mandatory components according to recommended guidance for surgery trials. This will allow comprehensive reporting of the interventions delivered during the trial.

- Due to the nature of the interventions, the trial personnel and participants are not blinded to treatment allocation.

MAIN TEXT

Background

Osteoarthritis (OA) is the commonest musculoskeletal condition that affects joints, causing pain, joint dysfunction and significant quality of life (QoL) impact. With rising obesity rates and ageing population, the number of people presenting with knee OA is increasing (1).

Patients with severe knee OA experiencing joint symptoms that substantially impact QoL are typically offered knee replacement (KR) to relieve pain and improve mobility. KR is clinically proven and cost-effective (2), however, artificial joints have a finite life span. If KR fails, revision is complex, costly and associated with higher morbidity, mortality, and inferior outcomes (3-5).

The James Lind Alliance established that defining the optimum timing of joint replacement, in order to achieve the best outcome is a significant patient concern(6). The number of young patients (55 years or less) undergoing KR is increasing (5), and risk of failure is disproportionately higher in the young and active. A combined endpoint analysis including revision, poor function and significant pain has shown KR success to be as low as 59% after 12-years in patients 60 years or less (7). Increasing life expectancy and the growing number of younger patients means there is a need for treatment which postpones the time to first KR, without compromising QoL or hampering ability to undergo KR at a later stage (7, 8). As in joint replacement in general, it is unknown whether treatment options preserving the joint are cost-effective (9, 10).

Knee Joint Distraction (KJD), the practice of placing an external fixator across a synovial joint and pulling the joint surfaces apart approximately 5mm for ~6 weeks, has been proposed as a potentially effective alternative to preserve the joint. The aim is to harness intrinsic joint-repair potential, providing cartilage repair and normalisation of subchondral bone abnormalities (11). KJD is not currently widely used in the UK, and no trials have been conducted in the NHS. Initial studies conducted in the Netherlands suggest it a safe and potentially effective treatment (12-14)One small trial suggested KJD to be non-inferior to total KR in function (15-17) and another predicted that it could save over 30% of revision KRs (18). With a willingness to pay €20,000 per Quality Adjusted Life Year (QALY), KJD was shown to be cost effective in over 75% cases for all age groups and over 90% in the young (55 years or less) (19).

For peer review only

Rationale

There is strong scientific basis for KJD with excellent cartilage regeneration in experimental OA with joint offloading procedures (20). Given the preliminary clinical data and underpinning science, KJD could be an alternative therapy to KR for younger patients, but current evidence is limited. Patient feedback highlighted the key priority for those in this age group is retaining their own knee, at expense of some residual knee pain. If KJD is shown to be safe, non-inferior to KR in terms of pain and cost-effective in the NHS then it could be routinely offered to patients 65 years or less, delaying need for KR and potentially avoiding revision surgery. This is the aim of the KARDS trial.

Methods and Design

Objectives

The primary objective is to conduct a multi-centre trial to investigate clinical effectiveness of KJD compared to KR in patients aged 65 years or less, with symptomatic knee OA severe enough to warrant KR, based on patient reported pain 12 months after surgery.

Secondary objectives are to investigate: 1) Patient reported outcomes; 2) Clinical outcomes of knee function; 3) Complications and need for further intervention; 4) Cost-effectiveness; 5) Participant experiences, intervention fidelity and barriers to wider implementation.

Trial design

This publication describes KARDS protocol V2.0, dated 29th September 2020.

KARDS is a phase III IDEAL Stage 3 Assessment (21), multi-centre, pragmatic, open-label, 1:1, two-arm individually randomised controlled trial, with embedded 12-month internal pilot phase.

The internal pilot phase will incorporate a qualitative process evaluation to identify potential barriers to recruitment and any challenges experienced in maintaining intervention fidelity. As part of the process evaluation, qualitative semi-structured interviews will be undertaken with clinicians, trial staff and participants to explore experiences of trial involvement and intervention acceptability during the pilot phase and throughout the main trial. Progression at the end of the pilot phase will be based on i) recruitment and dropout rates ii); safety; iii) the process evaluation.

<u>Trial setting and recruitment</u>

Participants will be recruited from secondary care orthopaedic centres following GP or specialist referral. Potentially eligible participants will be identified by the attending clinical team from orthopaedic outpatient

For peer review only

clinics and theatre lists. Following information provision, patients will be given the opportunity to discuss the trial with their family, friends and healthcare professionals before being invited to participate.

Informed consent will be obtained by the Principal Investigator (PI) or appropriate, delegated, healthcare professional as detailed on the Authorised Personnel Log, in accordance with the principles of Good Clinical Practice (GCP) and Declaration of Helsinki 1996.

All sites must be able to deliver both KR and KJD. As KJD is not a standard technique used in knee surgery not all surgeons will have the required experience, and some surgeons may not be in individual equipoise despite there being centre equipoise. A hybrid expertise-based design, where surgeons are categorised into “delivery units” based on experience, addresses both issues.

There are two delivery unit categories based on the interventions surgeons are authorised to perform within the trial: i) *Single delivery units* consist of surgeons authorised to deliver KJD **or** KR, where the surgeon performing the procedure will be chosen after randomisation, depending on the allocation, or ii) *Dual delivery unit*s, consisting surgeons authorised to deliver KJD **and** KR where a randomised participant may receive either operation by the same surgeon.

Eligibility

Surgeon eligibility: Participating surgeons must either be a consultant orthopaedic surgeon or perform the procedure under direct consultant supervision. To deliver KR within KARDS a surgeon must have performed ≥10 KRs in the past 12 months as the primary surgeon. To deliver KJD within KARDS they must have performed ≥10 external fixations during their career as the primary surgeon or completed a limb reconstruction fellowship.

Patient eligibility: criteria are minimised to ensure inclusivity and generalisability. Adult patients are eligible if aged ≤65 years requiring KR and meet the criteria in Table 1.

For peer review only

Table 1: Patient Eligibility Criteria

Inclusion Criteria	Exclusion Criteria
Age ≥18 years and ≤65 years at time of signing the Informed Consent form	Bone density not sufficient to support pins for 6 weeks*
Symptoms (pain and/or reduced function) severe enough to warrant knee replacement*	Isolated patella-femoral OA*
Pre-operative leg alignment not requiring correction*	Complete joint space obliteration in both medial and lateral tibio-femoral compartments as seen on weight bearing AP knee radiograph
Intact collateral knee ligaments*	A known diagnosis of inflammatory arthritis
Fixed flexion deformity ≤10[illegible]	Presence of a previous joint replacement in any limb
	Surgical treatment of involved knee within the past 6 months (excluding arthroscopy)
	Previous knee joint distraction on the involved knee
	Previously participated in the KARDS trial
	Weight >120kg
	Pregnant or lactating (confirmed by participant)
	Active cancer (currently diagnosed and under treatment)
	Unable to complete all trial procedures (e.g. attend follow up visits, complete questionnaires)
	Unable to provide informed consent (cognitive disorder such as dementia, psychiatric illness)

** In the opinion of the treating clinician*

For peer review only

Interventions

A surgical manual will document each trial procedure highlighting mandatory components according to recommended guidance (22).

Intervention (Knee Joint Distraction)

A definitive external fixator construct will be used which allows for controlled linear distraction across the knee joint of 5mm. The exact nature of the construct will depend on equipment availability at site and surgeon preference. Devices will be approved for trial use by the Trial Management Group

During surgery the external fixation frame will be assembled according to frame construct procedures detailed in the surgical manual, with focus on meticulous pin insertion to minimise complication risk. Pins will be placed under fluoroscopic control. Once assembly complete, ≥2mm and ≤5mm axial distraction will be applied across

the knee joint. A further 1mm distraction may be applied per day until 5mm distraction at the joint is confirmed radiographically, or up to 7 days.

External fixators will be removed under general or regional anaesthesia after six weeks. Local protocol for pin-site care will be followed and will be documented. Gentle manipulation under anaesthesia to achieve ≥90 degrees of motion will be attempted at the time of fixator removal.

Control (Knee replacement)

KR surgery will be performed in line with local practice and the surgical manual and will vary depending upon implant type and surgeon preference. Surgeons performing the procedure are expected to comply with specific surgical steps for the implant being used as detailed in the manufacturer instructions for use document.

Concomitant care and interventions

Pre-operative preparation and post-operative care will be provided to all trial participants in line with the site's usual protocol for KRs. Decisions about concomitant medications/treatments for symptomatic knee osteoarthritis will be according to local medical plan and clinical management. Details of analgesia and other medication prescribed will be collected throughout trial. Participants may require further intervention for symptomatic knee OA as per routine practice. Further clinical intervention is permitted for all participants and recorded for the trial.

Patient and Public Involvement

KARDS Patient and Public Involvement (PPI) group provided feedback on choice of primary outcome, minimally important difference used sample size calculations & the decision to not blind participants. PPI representatives on the Trial Management Group provided feedback on the schedule of events for participants.

<u>Randomisation and blinding</u>

Participants will be randomised into the trial by an authorised member of site staff, on a 1:1 basis between KJD and KR, based on a minimisation algorithm with random component balanced for delivery unit and OA severity (Kellgren-Lawrence Grades 2-3 vs. Grade 4) (23). Randomisation will be performed centrally using Leeds Clinical Trials Research Unit (CTRU) automated secure 24-hour randomisation web or telephone service, occurring on the same day as baseline visit, within 6 weeks of the planned surgery date. Clinical assessments and baseline questionnaires will be completed before randomisation with trial specific assessments performed afterwards. Treatment allocation will not be blinded to participants, medical staff, or clinical trial staff.

For peer review only

Data collection

Clinical data will be collected at baseline, day of surgery, prior to discharge, week 6 (KJD only), and months 3, 12, and 24 post-surgery. Participant completed data will be collected at baseline, day of surgery and months 3, 6, 12, and 24 post-surgery. Full assessment schedule based on the SPIRIT guidance (24) provided in Supplementary Material 1.

Participating sites will maintain a file of essential trial documentation including copies of all completed CRFs. Sites will post paper CRFs, and electronically transfer trial X-rays to Leeds CTRU. Trial data will be entered onto an electronic database, except post-surgery questionnaires completed using electronic remote data capture by participants or via postal questionnaire.

Data will be monitored for quality and completeness by CTRU. Missing data will be requested from sites until received, confirmed as unavailable or trial analysis begins. The sponsor reserves the right to conduct periodic source data verification to monitor trial integrity.

Participant qualitative interviews will be conducted by telephone, and staff interviews conducted in person, or telephone/video conference. Interviews will be audio recorded on an encrypted recorder, anonymised and transcribed verbatim for analysis.

All information collected during the trial will be kept strictly confidential. Information will be held securely on paper and electronically at Leeds CTRU, with process evaluation data held securely on Warwick CTU server. Both will comply with all aspects of the Data Protection Act 2018. If a participant withdraws consent from further trial treatment and/or further data collection, data to the point of withdrawal will remain on file and included in the analysis.

Outcome measures

Primary outcome measure

The primary outcome measure is Knee Injury and Osteoarthritis Outcomes Score (KOOS) pain score 12-months post-surgery. Pain was indicated by the PPI group as being the most important outcome to them. KOOS is a patient-administered questionnaire, validated for use in patients with knee OA or knee injury (25), recorded on a Likert Scale 0-4, transformed to 0 (worst) to 100 (best) scale.

Secondary outcome measures

1. *Patient report outcome measures (PROMs) and QoL within 24 months post-surgery*
    a. KOOS (overall and at component level)
    b. Pain Visual Analogue Scale (VAS) (26, 27)
    c. Oxford Knee Score (OKS) (28-30)
2. *Objective assessment of knee function*
    a. Active range of movement

For peer review only

    b. Timed-up-and-go test(31, 32)

3. *Incidence of complications, including infection*
    a. Intra-operative complications
    b. Post-operative complications(33)
4. *Further interventions within 24 months post-surgery*
    a. Further surgical interventions including conversion to KR or revision surgery
5. *KJD's potential as cartilage regenerative therapy*
    a. Joint space width (assessed using standardised fixed-flexion PA at 20° X-rays (34))
6. *Estimate of short- and long-term cost-effectiveness*
    a. EQ-5D-3L questionnaire at 24 months
    b. Health Resource Utilisation and Private Costs questionnaire at 24 months
    c. Incremental costs per Quality Adjusted Life Years (QALY) gained at 20 years
7. *Implementation processes and intervention fidelity*
    a. Quantitative *(*surgical CRF and central review of post-operative x-rays).
    b. Qualitative evaluation with surgical and clinical staff
8. *Qualitative evaluation of participant experiences*

Statistical Considerations and Analyses

Sample size

Power calculations are based on a non-inferiority hypothesis for the primary outcome measure, KOOS pain score. 344 participants (172 per arm) will have 90% power to demonstrate non-inferiority based on an 8 point non-inferiority margin, assuming a standard deviation of 21 points (2, 35-37), one-sided 2.5% significance level and 15% dropout rate. The non-inferiority margin was agreed by clinical and patient co-applicants based on being 33% less than the 12 point minimally important difference observed in previous trials (18, 38-40), clinical co-applicant experience and PPI focus group feedback. No adjustment has been made to accommodate surgeon learning curve since external fixation is a common procedure orthopaedic surgeons frequently do for trauma, and minimum expertise is required for surgeon eligibility.

Analysis methods

Full statistical analysis plan predefining all analyses and patient populations will be in place prior to any comparative analyses according to guidelines (41). KARDS will be reported according to the CONSORT extension for Non-Inferiority and Equivalence Randomized Trials (42). The intention-to-treat (ITT) population will include all randomised participants, and the per-protocol (PP) population will include all participants who received their randomised intervention as intended. Although there is no 'gold standard' for non-inferiority trials, outcomes will be analysed primarily for the PP population (43). A sensitivity analysis will be conducted for the ITT population.

For peer review only

The primary analysis will report adjusted estimates of treatment effect from multivariable regression of KOOS pain score at 12 months. Statistical significance of KJD non-inferiority relative to KR will be based on a 2-sided likelihood-based test with type 1 error of 2.5% in both tails, adjusted by baseline score and OA severity as fixed effects, and delivery unit as a random effect (44). If the 95% confidence interval for absolute difference in means between KJD and KR lies entirely below or includes the non-inferiority boundary then there would be insufficient evidence to reject the null hypothesis that KJD inferior to KR. Conversely, if the 95% confidence interval lies entirely above the non-inferiority boundary, there would be evidence to reject the null hypothesis and conclude KJD non-inferior to KR. If non-inferiority demonstrated and KJD appears superior to KR, based on estimated effect and associated confidence interval, statistical significance for superiority will be calculated based on an ITT analysis. Secondary analysis of the primary outcome measure will use multilevel modelling to account for longitudinal data collected over 24 months. Sensitivity analyses will be considered to investigate any impact of surgeon experience on treatment effect estimates(45).

Reasons for missing data will be examined and primary method to account for missing data will be chosen based on the most reasonable missing data mechanism assumption, with sensitivity analyses to assess robustness of results to different missing data mechanism assumptions.

Other PROM responses will be transformed into dimension scores, according to scoring manuals, and presented graphically and longitudinally. Standardised area under the curve (AUC) statistics will be compared across treatment groups as an analysis conditional on patient time in the trial. Functional assessments will be reported descriptively, along with joint space width for the KJD group.

Complications will be reported as unique events and unique patients experiencing events. Joint survival will be measured from randomisation to time of further intervention and analysed using the Kaplan Meier method.

Process evaluation interview data will be analysed using thematic content analysis to identify patterns or themes (46), using coding of audio-transcript recordings, adopting the framework method described by Ritchie and Spencer and Pope et al (47, 48). Normalisation Process Theory will be used as a theoretical framework to explore and explain extent of intervention implementation (49-51), using the software package NVivo 12 to manage data and facilitate this process. Interview data and full record of issues raised will be discussed in detail with the Trial Management Group and summarised for oversight committees. Good practice will be shared with other recruiting sites.

Cost-effectiveness analysis will be conducted from NHS and Personal Social Services perspectives and society over a 24-month time horizon. The analysis will estimate surgical intervention costs and primary and secondary health care services costs including complications, follow-up, medications and repeat medical procedures, and out of pocket and productivity costs to patients and their families. Outcomes will be evaluated using QALYs estimated by the AUC approach. Unit costs will be obtained from list prices for devices and materials involved in the interventions, medications list prices, NHS health professional staff salary scales,

For peer review only

primary care and community services opportunity costs (52), outpatient, inpatient admissions and Accident and Emergency visits NHS Reference Costs , and median UK gross hourly earnings(53). Generalised linear models will be used to adjust for unbalanced baseline covariates in costs (54, 55) and adjusting for baseline EQ-5D-3L score in analysing QALYs (56). Missing data will be imputed using established methods (57). Results will be presented in terms of incremental cost per QALY gained and cost per unit gain in 12-month KOOS. Sampling uncertainty will be analysed using the bootstrap method (58) and joint uncertainty in costs and QALYs will be analysed using cost-effectiveness acceptability curves (59).

A decision analytic model will be built to evaluate lifetime cost-effectiveness over 20 years by adapting and updating a published Markov model of delayed joint replacement using National Joint Registry, clinical study and UK life table data (9, 10). The model will account for trade-offs of delaying knee replacement in terms of reducing the risk of the patient requiring revision surgery near end of life and increased complication risk with primary operation at older age (9). Sampling uncertainty in model parameter values will be described using probabilistic sensitivity analysis, while key parameters affecting the likelihood of KJD meeting the NICE £20,000 threshold for cost-effectiveness (60) will be identified using Tornado plots.

Monitoring

An independent Trial Steering Committee (TSC), comprising a Statistician, two orthopaedic consultant surgeons and one patient representative, will have overall responsibility for trial oversight, monitoring trial progress, protocol adherence and participant safety. An independent Data Monitoring and Ethics Committee (DMEC) comprising a Statistician and two orthopaedic consultant surgeons, will review interim safety data by randomised group, reviewing the underlying statistical design assumptions to ensure the trial remains adequately powered. TSC and DMEC meetings will be conducted annually as a minimum according to agreed TSC and DMEC Charters (61).

No formal guidelines for stopping the trial early are in place since no formal planned interim analysis of the primary outcome is planned.

Information on complications will be collected from randomisation to end of trial defined as the last visit date of the last patient. Serious complications will be subject to expedited reporting where sites will inform CTRU within 24 hours of becoming aware of it. Suspected or confirmed pregnancies and all deaths from randomisation until the end of trial will be reported to CTRU.

Ethics and Dissemination

For peer review only

KARDS is funded by NIHR HTA (reference: 17/122/06) and sponsored by the University of Leeds, approved by the Research Ethics Committee (REC) (reference: 19/YH/0368) and Health Research Authority (HRA). All amendments will be submitted for approval and communicated to sites in accordance with HRA guidelines.

Trial results will be disseminated at relevant clinical conferences and societies, published in peer-reviewed journals and disseminated through relevant patient groups. Authorship will be according to ICMJE guidelines.

Discussion

KARDS is a pragmatic, multi-centre prospective randomised controlled trial conducted in an NHS setting, the aim is to determine if KJD is non-inferior to KR in terms of pain and cost-effective in the NHS then it could be routinely offered to patients aged 65 years or less. In addition, it will report on radiological outcomes and patient acceptability. It will be a definitive IDEAL Stage 3 (Assessment) trial (21) with potential to lead to a paradigm shift if it demonstrates non-inferiority of KJD compared to KR.

Joint distraction outcomes at various anatomical locations have been reported in several case series. Though small numbers of patients have been involved, results are encouraging in at least providing temporary symptom relief. At the ankle, improvements in reported symptoms were seen in 73-91% of patients at mean follow up time of 1-12 years (62). Joint distraction has been demonstrated to give good clinical outcomes in first carpometacarpal joint osteoarthritis, albeit in a very limited number of patients. Patients were followed for 1 year with improved functional scores compared to baseline (63). The KJD literature is difficult to assess due to heterogeneity of devices and methods used. A recent review included one cohort study and two small trials all of which came from the same research group including a total of 62 patients (64). These studies all utilised a spring-loaded static distractor. Western Ontario and McMaster Universities Osteoarthritis Index score improvements were significantly greater one-year post KJD than conservatively managed osteoarthritis (17), and not inferior to total KR (19), or high tibial osteotomy (HTO) (11).

Two studies (11, 19) reported KOOS, Intermittent and Constant Osteoarthritis Pain score, EuroQol 5 Dimensions (EQ-5D) and Short Form (SF)-36 with significant improvements at one year seen in all scores except for the SF-36 mental component score, with no significant difference in these improvements compared to KR or HTO. Pain score assessed on pain VAS was reported in both studies and showed improvements at one year with no significant difference between KJD and HTO or KR. Radiographic assessment of joint structure has been undertaken in various studies, with imaging at the time of distraction or follow up. The group above utilised MRI to assess structural recovery. Mean cartilage thickness was shown to increase on both the tibial and femoral sides and percentage of joint surface appearing as denuded subchondral bone decreased (64). Radiographic minimum joint space width was shown to increase by 0.8mm at 12 months compared to baseline (11, 17, 19), Similar to another study where the mean joint space width, measured using standardised digital techniques, increased from 2.7mm to 3.6mm 12 months post-fixator removal (65).

For peer review only

The most frequently reported KJD complication is pin site infection. Rates approaching 70% have been reported, with 20% of affected patients requiring intravenous therapy (64). In the series of 62 patients described above, two patients required surgical intervention for pin-site infection during distraction, with a further case of osteomyelitis requiring surgery following fixator removal (11, 17, 19). These infection rates are at odds with those reported in patients treated by definitive external fixation for other reasons. Pin-site infection rates of 40% are found fairly consistently, even where fixators are in place for much longer, the reasons for this are unclear (66). Whilst transient pin-site infection seldom has long term implications, it is unpleasant for patients and may impair rehabilitation. Deep infections may be more worrisome, especially considering expected osteoarthritis progression following distraction potentially requiring eventual arthroplasty. Wherever possible, external fixator pins will be sited outside the implantation zone of a KR. Total KR following significant osteomyelitis is significantly more complex and has further infection risk even when infection considered eradicated (67). Current KJD literature does not provide sufficient evidence to estimate serious infection rates following conversion to KR. In one ankle distraction study with over five years follow up, there was no infection seen in five patients who had conversion to arthroplasty (68). Loss of knee range of movement immediately following distraction therapy has been observed to return after 1 year, with a small number of patients undergoing joint manipulation under anaesthetic to achieve this (13, 19).

Trial strengths include its pragmatic nature, standardised surgical and assessment techniques, robust reporting and safety mechanisms and appropriate sample size. The window of six weeks between baseline measures and planned surgery date aligns with clinical pathways and ensures recruitment feasibility. KARDS has a pragmatic hybrid expertise-based design, where surgeons are categorised into "delivery units" based on their experience, a successful approach successful for similar knee OA surgical trials (69). Furthermore, clinicians are free to choose KR implant type and KJD external fixator. This choice brings a limitation in not being able to determine potential individual mechanisms of action limiting individual indications and/or contraindications. Those implants and fixators approved in the trial protocol are based upon consensus amongst experts and published literature. Sub-group analysis will not be adequately powered to determine if a particular fixator type is superior. A further limitation is the lack of blinding but this is unavoidable. It would be impractical to blind medical staff prior to surgery at many sites as they need to plan for the specific surgery. PPI feedback was that being blinded until just before or after surgery would be unacceptable if the medical team knew the allocation. The primary outcome measure is patient reported and therefore it is not possible to have a blinded primary outcome assessment.

Declaration of interests

HKS – Consultant to Orthofix and received research grant from Orthofix. Received payment for teaching responsibilities from Orthofix and Smith & Nephew.

For peer review only

HP - received grant funding from KTP, Pacira Pharmaceuticals, Zimmer Biomet Healthcare, B Braun & Welcome Trust. Received consulting fees from Medacta International, Smith and Nephew, Depuy Synthes, JRI Orthopaedics, Janssen, Meril Life, Zimmer Biomet & Paradigm Pharmaceuticals. Received payment from Invibio for presentations, from Kennedy's Law for expert testimony & from Pacira Pharmaceuticals for study conduct. Received payments from Medacta International, Depuy Synthes & Zimmer Biomet for attending meetings/travel.

HSi - received grant funding from EPSRC Ultrasonic Surgery & EPSRC 2050 EnLightenus. Submitted patent with Joint Assist patient application.

AM – received grant funding for from Stryker for the RACER-Hip trial.

Authors' contributions

KARDS study design: RH, IS, DDS, HP, CT, AM, HSi, DM, DMc, HKS, TWH, DE, CF, JC, PJH, CR. Writing of manuscript: RH, CT, IS, DDS, HP, SA, PJH, RMM, HSi. All authors have read and approved of the final manuscript. Acknowledgement of RL for contributions to the KARDS study design.

Funding

This study is funded by the National Institute for Health Research (NIHR) Health Technology Assessment (HTA) Programme (Project: 17/122/06). The views expressed are those of the authors and not necessarily those of the NIHR or Department of Health and Social Care.

Access to data

To maintain scientific integrity of the trial, outcome data will not be released prior to publication of the analysis of the primary outcome measure, either for trial publication or oral presentation purposes, without permission of the TSC. Following publication, requests for sharing of trial data may be submitted to Leeds CTRU.

References

1. Lawrence RC, Felson DT, Helmick CG, Arnold LM, Choi H, Deyo RA, et al. Estimates of the prevalence of arthritis and other rheumatic conditions in the United States. Part II. Arthritis and rheumatism. 2008;58(1):26-35.
2. Skou ST, Roos EM, Laursen MB, Rathleff MS, Arendt-Nielsen L, Simonsen O, et al. A Randomized, Controlled Trial of Total Knee Replacement. The New England journal of medicine. 2015;373(17):1597-606.
3. Bayliss LE, Culliford D, Monk AP, Glyn-Jones S, Prieto-Alhambra D, Judge A, et al. The effect of patient age at intervention on risk of implant revision after total replacement of the hip or knee: a population-based cohort study. Lancet (London, England). 2017;389(10077):1424-30.
4. Deehan DJ, Murray JD, Birdsall PD, Pinder IM. Quality of life after knee revision arthroplasty. Acta orthopaedica. 2006;77(5):761-6.
5. Stambough JB, Clohisy JC, Barrack RL, Nunley RM, Keeney JA. Increased risk of failure following revision total knee replacement in patients aged 55 years and younger. The bone & joint journal. 2014;96-b(12):1657-62.

For peer review only

6. About the James Lind Alliance: James Lind Alliance [Internet]; [cited 2018 Jul 25] [Available from: http://www.jla.nihr.ac.uk/about-the-james-lind-alliance/.
7. Price AJ, Longino D, Rees J, Rout R, Pandit H, Javaid K, et al. Are pain and function better measures of outcome than revision rates after TKR in the younger patient? The Knee. 2010;17(3):196-9.
8. Bitton R. The economic burden of osteoarthritis. The American journal of managed care. 2009;15(8 Suppl):S230-5.
9. Mota RE. Cost-effectiveness analysis of early versus late total hip replacement in Italy. Value Health. 2013;16(2):267-79.
10. Lan RH, Yu J, Samuel LT, Pappas MA, Brooks PJ, Kamath AFJTJoA. How are we measuring cost-effectiveness in total joint arthroplasty studies? Systematic review of the literature. 2020;35(11):3364-74.
11. van der Woude JAD, Wiegant K, van Heerwaarden RJ, Spruijt S, van Roermund PM, Custers RJH, et al. Knee joint distraction compared with high tibial osteotomy: a randomized controlled trial. Knee Surg Sports Traumatol Arthrosc. 2017;25(3):876-86.
12. Jansen MP, Besselink NJ, van Heerwaarden RJ, Custers RJH, Emans PJ, Spruijt S, et al. Knee Joint Distraction Compared with High Tibial Osteotomy and Total Knee Arthroplasty: Two-Year Clinical, Radiographic, and Biochemical Marker Outcomes of Two Randomized Controlled Trials. Cartilage. 2021;12(2):181-91.
13. Jansen MP, Mastbergen SC, van Heerwaarden RJ, Spruijt S, van Empelen MD, Kester EC, et al. Knee joint distraction in regular care for treatment of knee osteoarthritis: A comparison with clinical trial data. PLoS One. 2020;15(1):e0227975.
14. Wiegant K, van Roermund PM, Intema F, Cotofana S, Eckstein F, Mastbergen SC, et al. Sustained clinical and structural benefit after joint distraction in the treatment of severe knee osteoarthritis. Osteoarthritis and cartilage. 2013;21(11):1660-7.
15. Bellamy N, Buchanan WW, Goldsmith CH, Campbell J, Stitt LW. Validation study of WOMAC: a health status instrument for measuring clinically important patient relevant outcomes to antirheumatic drug therapy in patients with osteoarthritis of the hip or knee. The Journal of rheumatology. 1988;15(12):1833-40.
16. Lequesne M. Indices of severity and disease activity for osteoarthritis. Seminars in arthritis and rheumatism. 1991;20(6 Suppl 2):48-54.
17. van der Woude JAD, Wiegant K, van Roermund PM, Intema F, Custers RJH, Eckstein F, et al. Five-Year Follow-up of Knee Joint Distraction: Clinical Benefit and Cartilaginous Tissue Repair in an Open Uncontrolled Prospective Study. Cartilage. 2017;8(3):263-71.
18. van der Woude JAD, Wiegant K, van Heerwaarden RJ, Spruijt S, Emans PJ, Mastbergen SC, et al. Knee joint distraction compared with total knee arthroplasty. 2017;99-B(1):51-8.
19. van der Woude JAD, Nair SC, Custers RJH, van Laar JM, Kuchuck NO, Lafeber FP, et al. Knee Joint Distraction Compared to Total Knee Arthroplasty for Treatment of End Stage Osteoarthritis: Simulating Long-Term Outcomes and Cost-Effectiveness. PLoS One. 2016;11(5):e0155524.
20. Wiegant K, Intema F, van Roermund PM, Barten-van Rijbroek AD, Doornebal A, Hazewinkel HA, et al. Evidence of cartilage repair by joint distraction in a canine model of osteoarthritis. Arthritis & rheumatology (Hoboken, NJ). 2015;67(2):465-74.
21. Hirst A, Philippou Y, Blazeby J, Campbell B, Campbell M, Feinberg J, et al. No Surgical Innovation Without Evaluation: Evolution and Further Development of the IDEAL Framework and Recommendations. 2019;269(2):211-20.
22. Blencowe N, Mills N, Cook J, Donovan J, Rogers C, Whiting P, et al. Standardizing and monitoring the delivery of surgical interventions in randomized clinical trials. 2016;103(10):1377-84.
23. Kellgren JH, Lawrence JS. Radiological assessment of osteo-arthrosis. Annals of the rheumatic diseases. 1957;16(4):494-502.

For peer review only

24. Chan A-W, Tetzlaff JM, Gøtzsche PC, Altman DG, Mann H, Berlin JA, et al. SPIRIT 2013 explanation and elaboration: guidance for protocols of clinical trials. BMJ : British Medical Journal. 2013;346(jan08 15):e7586-e.
25. Rolfson O, Wissig S, van Maasakkers L, Stowell C, Ackerman I, Ayers D, et al. Defining an International Standard Set of Outcome Measures for Patients With Hip or Knee Osteoarthritis: Consensus of the International Consortium for Health Outcomes Measurement Hip and Knee Osteoarthritis Working Group. Arthritis care & research. 2016;68(11):1631-9.
26. Hawker GA, Mian S, Kendzerska T, French M. Measures of adult pain: Visual Analog Scale for Pain (VAS Pain), Numeric Rating Scale for Pain (NRS Pain), McGill Pain Questionnaire (MPQ), Short-Form McGill Pain Questionnaire (SF-MPQ), Chronic Pain Grade Scale (CPGS), Short Form-36 Bodily Pain Scale (SF-36 BPS), and Measure of Intermittent and Constant Osteoarthritis Pain (ICOAP). Arthritis care & research. 2011;63 Suppl 11:S240-52.
27. McCormack HM, Horne DJ, Sheather S. Clinical applications of visual analogue scales: a critical review. Psychological medicine. 1988;18(4):1007-19.
28. Dawson J, Fitzpatrick R, Murray D, Carr A. Questionnaire on the perceptions of patients about total knee replacement. The Journal of bone and joint surgery British volume. 1998;80(1):63-9.
29. Dunbar MJ, Robertsson O, Ryd L, Lidgren L. Appropriate questionnaires for knee arthroplasty. Results of a survey of 3600 patients from The Swedish Knee Arthroplasty Registry. The Journal of bone and joint surgery British volume. 2001;83(3):339-44.
30. Xie F, Ye H, Zhang Y, Liu X, Lei T, Li SC. Extension from inpatients to outpatients: validity and reliability of the Oxford Knee Score in measuring health outcomes in patients with knee osteoarthritis. International journal of rheumatic diseases. 2011;14(2):206-10.
31. CDC. Timed Up & Go [Available from: https://www.cdc.gov/steadi/pdf/TUG_test-print.pdf.
32. Davis AM, King LK, Stanaitis I, Hawker GAJO, Cartilage. Fundamentals of osteoarthritis: outcome evaluation with patient-reported measures and functional tests. 2021.
33. GOV.UK. Surgical site infection surveillance service: protocol and guides [Internet] [cited 2019 Sep 9] [Available from: https://www.gov.uk/government/publications/surgical-site-infection-surveillance-service-protocol-procedure-codes-and-user-manual.
34. Marijnissen AC, Vincken KL, Vos PA, Saris DB, Viergever MA, Bijlsma JW, et al. Knee Images Digital Analysis (KIDA): a novel method to quantify individual radiographic features of knee osteoarthritis in detail. Osteoarthritis and cartilage. 2008;16(2):234-43.
35. A WD, Toksvig-Larsen S, Roos EM. Association between knee alignment and knee pain in patients surgically treated for medial knee osteoarthritis by high tibial osteotomy. A one year follow-up study. BMC musculoskeletal disorders. 2009;10:154.
36. Nilsdotter AK, Toksvig-Larsen S, Roos EM. Knee arthroplasty: are patients' expectations fulfilled? A prospective study of pain and function in 102 patients with 5-year follow-up. Acta orthopaedica. 2009;80(1):55-61.
37. Roos EM, Toksvig-Larsen S. Knee injury and Osteoarthritis Outcome Score (KOOS) - validation and comparison to the WOMAC in total knee replacement. Health and quality of life outcomes. 2003;1:17.
38. State of Musculoskeletal Health 2017: Arthritis Research UK; 2017 [
39. Collins NJ, Misra D, Felson DT, Crossley KM, Roos EM. Measures of knee function: International Knee Documentation Committee (IKDC) Subjective Knee Evaluation Form, Knee Injury and Osteoarthritis Outcome Score (KOOS), Knee Injury and Osteoarthritis Outcome Score Physical Function Short Form (KOOS-PS), Knee Outcome Survey Activities of Daily Living Scale (KOS-ADL), Lysholm Knee Scoring Scale, Oxford Knee Score (OKS), Western Ontario and McMaster Universities Osteoarthritis Index (WOMAC), Activity Rating Scale (ARS), and Tegner Activity Score (TAS). Arthritis care & research. 2011;63 Suppl 11(0 11):S208-28.
40. Devji T, Guyatt GH, Lytvyn L, Brignardello-Petersen R, Foroutan F, Sadeghirad B, et al. Application of minimal important differences in degenerative knee disease outcomes: a

For peer review only

systematic review and case study to inform BMJ Rapid Recommendations. 2017;7(5):e015587.
41. Gamble C, Krishan A, Stocken D, Lewis S, Juszczak E, Doré C, et al. Guidelines for the Content of Statistical Analysis Plans in Clinical Trials. Jama. 2017;318(23):2337-43.
42. Piaggio G, Elbourne DR, Pocock SJ, Evans SJ, Altman DG. Reporting of noninferiority and equivalence randomized trials: extension of the CONSORT 2010 statement. Jama. 2012;308(24):2594-604.
43. Rehal S, Morris TP, Fielding K, Carpenter JR, Phillips PP. Non-inferiority trials: are they inferior? A systematic review of reporting in major medical journals. BMJ open. 2016;6(10):e012594.
44. Beard DJ, Davies LJ, Cook JA, MacLennan G, Price A, Kent S, et al. The clinical and cost-effectiveness of total versus partial knee replacement in patients with medial compartment osteoarthritis (TOPKAT): 5-year outcomes of a randomised controlled trial. The Lancet. 2019;394(10200):746-56.
45. Corrigan N, Marshall H, Croft J, Copeland J, Jayne D, Brown J. Exploring and adjusting for potential learning effects in ROLARR: a randomised controlled trial comparing robotic-assisted vs. standard laparoscopic surgery for rectal cancer resection. Trials. 2018;19(1):339.
46. Braun V, Clarke V. Using thematic analysis in psychology. Qualitative Research in Psychology. 2006;3(2):77-101.
47. NICE. Guide to the methods of technology appraisal 2013. NICE; 2013.
48. Pope C, Mays N. Reaching the parts other methods cannot reach: an introduction to qualitative methods in health and health services research. BMJ (Clinical research ed). 1995;311(6996):42-5.
49. May C, Finch T. Implementing, Embedding, and Integrating Practices: An Outline of Normalization Process Theory. 2009;43(3):535-54.
50. May C, Finch T, Mair F, Ballini L, Dowrick C, Eccles M, et al. Understanding the implementation of complex interventions in health care: the normalization process model. BMC Health Services Research. 2007;7(1):148.
51. May CR, Mair F, Finch T, MacFarlane A, Dowrick C, Treweek S, et al. Development of a theory of implementation and integration: Normalization Process Theory. Implementation Science. 2009;4(1):29.
52. Curtis L, Burns A. Unit Costs of Health and Social Care 2020, Personal Social Services Research Unit, University of Kent, Canterbury. 2020.
53. Statistics OfN. Annual Survey of Hours and Earnings (ASHE) 2020. [Available from: https://www.ons.gov.uk/employmentandlabourmarket/peopleinwork/earningsandworkinghours/
54. Mihaylova B, Briggs A, O'Hagan A, Thompson SG. Review of statistical methods for analysing healthcare resources and costs. Health economics. 2011;20(8):897-916.
55. Thompson SG, Nixon RM. How sensitive are cost-effectiveness analyses to choice of parametric distributions? Medical decision making : an international journal of the Society for Medical Decision Making. 2005;25(4):416-23.
56. Manca A, Hawkins N, Sculpher MJ. Estimating mean QALYs in trial-based cost-effectiveness analysis: the importance of controlling for baseline utility. Health economics. 2005;14(5):487-96.
57. Faria R, Gomes M, Epstein D, White IR. A guide to handling missing data in cost-effectiveness analysis conducted within randomised controlled trials. PharmacoEconomics. 2014;32(12):1157-70.
58. Briggs AH, Wonderling DE, Mooney CZ. Pulling cost-effectiveness analysis up by its bootstraps: a non-parametric approach to confidence interval estimation. Health economics. 1997;6(4):327-40.
59. Fenwick E, Claxton K, Sculpher M. Representing uncertainty: the role of cost-effectiveness acceptability curves. Health economics. 2001;10(8):779-87.

For peer review only

60. National Institute for H, Care E. NICE Process and Methods Guides. Guide to the Methods of Technology Appraisal 2013. London: National Institute for Health and Care Excellence (NICE)

Copyright © 2013 National Institute for Health and Clinical Excellence, unless otherwise stated. All rights reserved.; 2013.
61. A proposed charter for clinical trial data monitoring committees: helping them to do their job well. Lancet (London, England). 2005;365(9460):711-22.
62. van Valburg AA, van Roermund PM, Lammens J, van Melkebeek J, Verbout AJ, Lafeber EP, et al. Can Ilizarov joint distraction delay the need for an arthrodesis of the ankle? A preliminary report. The Journal of bone and joint surgery British volume. 1995;77(5):720-5.
63. Spaans AJ, Minnen LPV, Braakenburg A, Mink van der Molen AB. Joint distraction for thumb carpometacarpal osteoarthritis: a feasibility study with 1-year follow-up. Journal of plastic surgery and hand surgery. 2017;51(4):254-8.
64. Takahashi T, Baboolal TG, Lamb J, Hamilton TW, Pandit HG. Is Knee Joint Distraction a Viable Treatment Option for Knee OA?-A Literature Review and Meta-Analysis. The journal of knee surgery. 2019;32(8):788-95.
65. Intema F, Van Roermund PM, Marijnissen AC, Cotofana S, Eckstein F, Castelein RM, et al. Tissue structure modification in knee osteoarthritis by use of joint distraction: an open 1-year pilot study. Annals of the rheumatic diseases. 2011;70(8):1441-6.
66. Ferguson D, Harwood P, Allgar V, Roy A, Foster P, Taylor M, et al. The PINS Trial: a prospective randomized clinical trial comparing a traditional versus an emollient skincare regimen for the care of pin-sites in patients with circular frames. The bone & joint journal. 2021;103-b(2):279-85.
67. Williams RL, Khan W, Roberts-Huntleigh N, Morgan-Jones R. Total knee arthroplasty in patients with prior adjacent multi-organism osteomyelitis. Acta orthopaedica Belgica. 2018;84(2):184-91.
68. Nguyen MP, Pedersen DR, Gao Y, Saltzman CL, Amendola A. Intermediate-term follow-up after ankle distraction for treatment of end-stage osteoarthritis. The Journal of bone and joint surgery American volume. 2015;97(7):590-6.
69. Beard D, Price A, Cook J, Fitzpatrick R, Carr A, Campbell M, et al. Total or Partial Knee Arthroplasty Trial - TOPKAT: study protocol for a randomised controlled trial. Trials. 2013;14(1):292.

For peer review only

	Screening and Consent	Randomisation & Baseline		Surgery (Day 0)	Follow-up time point						Unscheduled
					Clinic visits			Postal questionnaire packs	Clinic visits		
	Prior to registration	Before randomisation and within 6 weeks prior to surgery	After randomisation and within 6 weeks prior to surgery		Post-operative (Up to Day 7)	Fixator Removal (Week 6)	Follow up (Month 3)	Follow up (Month 6)	Follow up (Month 12)	Follow up (Month 24)	
	All	All	All	All	All	KJD arm only	All	All	All	All	All
Informed Consent	X										
Screening Data	X										
Eligibility	X										
Patient Details	X										
Patient Demographics		X									
Medical History		X									
OA Severity (Kellgren-Lawrence grade based on standard AP & lateral x-rays)		X									
Physical examination of knee		X									
TUG (Timed up and go test)		X					X		X	X	
ROM (Range of movement) using goniometer		X					X		X	X	
Rosenberg View X-ray			X				X^		X^	X^	
Surgery (KR or KJD)				X							
Surgical details				X		X^					
Distraction of external fixator (KJD only)				X^	X^						
Removal of external fixator (KJD only)						X^					
Intra-operative Complications				X		X^					
Additional knee related and/or other limb surgery				X	X	X^	X	X	X	X	X
Concomitant Medications				X	X	X^	X	X	X	X	X
Discharge Details					X	X^					

For peer review only

AP/Lateral View X-rays					X	X^					
Post-operative Complications					X	X^	X	X	X	X	X
**Patient Reported Outcomes**											
KOOS		X		X*			X	X	X	X	
OKS		X					X	X	X	X	
EQ5D-3L		X					X	X	X	X	
Pain VAS		X					X	X	X	X	
Health Resource Use		X					X	X	X	X	
Serious complications											X
Participant withdrawal											X
Re-operation											X
Pregnancy											X
Death											X

*Up to 1 day before surgery
^KJD arm only

For peer review only

SPIRIT 2013 Checklist: Recommended items to address in a clinical trial protocol and related documents*

Section/item	Item No	Description	Addressed on page number
**Administrative information**			
Title	1	Descriptive title identifying the study design, population, interventions, and, if applicable, trial acronym	______1______
Trial registration	2a	Trial identifier and registry name. If not yet registered, name of intended registry	______2______
	2b	All items from the World Health Organization Trial Registration Data Set	______11_____
Protocol version	3	Date and version identifier	______4______
Funding	4	Sources and types of financial, material, and other support	______11_____
Roles and responsibilities	5a	Names, affiliations, and roles of protocol contributors	______1______
	5b	Name and contact information for the trial sponsor	______11_____
	5c	Role of study sponsor and funders, if any, in study design; collection, management, analysis, and interpretation of data; writing of the report; and the decision to submit the report for publication, including whether they will have ultimate authority over any of these activities	____11-12____
	5d	Composition, roles, and responsibilities of the coordinating centre, steering committee, endpoint adjudication committee, data management team, and other individuals or groups overseeing the trial, if applicable (see Item 21a for data monitoring committee)	______11_____

For peer review only

Introduction

Background and rationale	6a	Description of research question and justification for undertaking the trial, including summary of relevant studies (published and unpublished) examining benefits and harms for each intervention	_____3-4_____
	6b	Explanation for choice of comparators	______3______
Objectives	7	Specific objectives or hypotheses	______4______
Trial design	8	Description of trial design including type of trial (eg, parallel group, crossover, factorial, single group), allocation ratio, and framework (eg, superiority, equivalence, noninferiority, exploratory)	______4______

Methods: Participants, interventions, and outcomes

Study setting	9	Description of study settings (eg, community clinic, academic hospital) and list of countries where data will be collected. Reference to where list of study sites can be obtained	_____4-5_____
Eligibility criteria	10	Inclusion and exclusion criteria for participants. If applicable, eligibility criteria for study centres and individuals who will perform the interventions (eg, surgeons, psychotherapists)	______5-6_____
Interventions	11a	Interventions for each group with sufficient detail to allow replication, including how and when they will be administered	______6-7_____
	11b	Criteria for discontinuing or modifying allocated interventions for a given trial participant (eg, drug dose change in response to harms, participant request, or improving/worsening disease)	______NA_____
	11c	Strategies to improve adherence to intervention protocols, and any procedures for monitoring adherence (eg, drug tablet return, laboratory tests)	6; surgical manual 5; hybrid-expertise design
	11d	Relevant concomitant care and interventions that are permitted or prohibited during the trial	______7______
Outcomes	12	Primary, secondary, and other outcomes, including the specific measurement variable (eg, systolic blood pressure), analysis metric (eg, change from baseline, final value, time to event), method of aggregation (eg, median, proportion), and time point for each outcome. Explanation of the clinical relevance of chosen efficacy and harm outcomes is strongly recommended	_____8-9_____

For peer review only - http://bmjopen.bmj.com/site/about/guidelines.xhtml

Participant timeline	13	Time schedule of enrolment, interventions (including any run-ins and washouts), assessments, and visits for participants. A schematic diagram is highly recommended (see Figure)	______8______
Sample size	14	Estimated number of participants needed to achieve study objectives and how it was determined, including clinical and statistical assumptions supporting any sample size calculations	______9_____
Recruitment	15	Strategies for achieving adequate participant enrolment to reach target sample size	4 & 8; pilot phase & qualitative process

Methods: Assignment of interventions (for controlled trials)

Allocation:

Sequence generation	16a	Method of generating the allocation sequence (eg, computer-generated random numbers), and list of any factors for stratification. To reduce predictability of a random sequence, details of any planned restriction (eg, blocking) should be provided in a separate document that is unavailable to those who enrol participants or assign interventions	______7______
Allocation concealment mechanism	16b	Mechanism of implementing the allocation sequence (eg, central telephone; sequentially numbered, opaque, sealed envelopes), describing any steps to conceal the sequence until interventions are assigned	______7______
Implementation	16c	Who will generate the allocation sequence, who will enrol participants, and who will assign participants to interventions	______7______
Blinding (masking)	17a	Who will be blinded after assignment to interventions (eg, trial participants, care providers, outcome assessors, data analysts), and how	______7______
	17b	If blinded, circumstances under which unblinding is permissible, and procedure for revealing a participant's allocated intervention during the trial	______NA______

Methods: Data collection, management, and analysis

For peer review only - http://bmjopen.bmj.com/site/about/guidelines.xhtml

Data collection methods	18a	Plans for assessment and collection of outcome, baseline, and other trial data, including any related processes to promote data quality (eg, duplicate measurements, training of assessors) and a description of study instruments (eg, questionnaires, laboratory tests) along with their reliability and validity, if known. Reference to where data collection forms can be found, if not in the protocol	_____8-9______
	18b	Plans to promote participant retention and complete follow-up, including list of any outcome data to be collected for participants who discontinue or deviate from intervention protocols	______8______
Data management	19	Plans for data entry, coding, security, and storage, including any related processes to promote data quality (eg, double data entry; range checks for data values). Reference to where details of data management procedures can be found, if not in the protocol	______8______
Statistical methods	20a	Statistical methods for analysing primary and secondary outcomes. Reference to where other details of the statistical analysis plan can be found, if not in the protocol	_____9-11_____
	20b	Methods for any additional analyses (eg, subgroup and adjusted analyses)	_____9-10_____
	20c	Definition of analysis population relating to protocol non-adherence (eg, as randomised analysis), and any statistical methods to handle missing data (eg, multiple imputation)	______9______
**Methods: Monitoring**			
Data monitoring	21a	Composition of data monitoring committee (DMC); summary of its role and reporting structure; statement of whether it is independent from the sponsor and competing interests; and reference to where further details about its charter can be found, if not in the protocol. Alternatively, an explanation of why a DMC is not needed	______11_____
	21b	Description of any interim analyses and stopping guidelines, including who will have access to these interim results and make the final decision to terminate the trial	______11_____
Harms	22	Plans for collecting, assessing, reporting, and managing solicited and spontaneously reported adverse events and other unintended effects of trial interventions or trial conduct	______11_____
Auditing	23	Frequency and procedures for auditing trial conduct, if any, and whether the process will be independent from investigators and the sponsor	______11_____
**Ethics and dissemination**			

For peer review only - http://bmjopen.bmj.com/site/about/guidelines.xhtml

Research ethics approval	24	Plans for seeking research ethics committee/institutional review board (REC/IRB) approval	_______11_____
Protocol amendments	25	Plans for communicating important protocol modifications (eg, changes to eligibility criteria, outcomes, analyses) to relevant parties (eg, investigators, REC/IRBs, trial participants, trial registries, journals, regulators)	_______12_____
Consent or assent	26a	Who will obtain informed consent or assent from potential trial participants or authorised surrogates, and how (see Item 32)	_______5______
	26b	Additional consent provisions for collection and use of participant data and biological specimens in ancillary studies, if applicable	_______NA_____
Confidentiality	27	How personal information about potential and enrolled participants will be collected, shared, and maintained in order to protect confidentiality before, during, and after the trial	_______5______
Declaration of interests	28	Financial and other competing interests for principal investigators for the overall trial and each study site	_____13-14_____
Access to data	29	Statement of who will have access to the final trial dataset, and disclosure of contractual agreements that limit such access for investigators	_______14_____
Ancillary and post-trial care	30	Provisions, if any, for ancillary and post-trial care, and for compensation to those who suffer harm from trial participation	_______NA_____
Dissemination policy	31a	Plans for investigators and sponsor to communicate trial results to participants, healthcare professionals, the public, and other relevant groups (eg, via publication, reporting in results databases, or other data sharing arrangements), including any publication restrictions	_______13______
	31b	Authorship eligibility guidelines and any intended use of professional writers	_______14______
	31c	Plans, if any, for granting public access to the full protocol, participant-level dataset, and statistical code	_______14______
**Appendices**			
Informed consent materials	32	Model consent form and other related documentation given to participants and authorised surrogates	________NA_____

For peer review only - http://bmjopen.bmj.com/site/about/guidelines.xhtml

Biological specimens	33	Plans for collection, laboratory evaluation, and storage of biological specimens for genetic or molecular analysis in the current trial and for future use in ancillary studies, if applicable	________NA______

*It is strongly recommended that this checklist be read in conjunction with the SPIRIT 2013 Explanation & Elaboration for important clarification on the items. Amendments to the protocol should be tracked and dated. The SPIRIT checklist is copyrighted by the SPIRIT Group under the Creative Commons "Attribution-NonCommercial-NoDerivs 3.0 Unported" license.

For peer review only
