# Supplementary material for: Clinical and cost-effectiveness of Knee Arthroplasty versus Joint Distraction for Osteoarthritis (KARDS): protocol for a multicentre, phase III, randomised control trial
Source: BMJ Open. 2022 Jun 30;12(6):e062721. doi: 10.1136/bmjopen-2022-062721 (PMC9247693; doi:10.1136/bmjopen-2022-062721)
Supplement: Reviewer comments [file bmjopen-2022-062721.reviewer_comments.pdf]

PEER REVIEW HISTORY

BMJ Open publishes all reviews undertaken for accepted manuscripts. Reviewers are asked to complete a checklist review form (**http://bmjopen.bmj.com/site/about/resources/checklist.pdf**) and are provided with free text boxes to elaborate on their assessment. These free text comments are reproduced below.

ARTICLE DETAILS

**TITLE (PROVISIONAL)**	Clinical and Cost-effectiveness of Knee Arthroplasty versus Joint Distraction for Osteoarthritis (KARDS): Protocol for A Multicentre, Phase III, Randomised Control Trial
**AUTHORS**	Tassinari, Cerys; Higham, Ruchi; Smith, Isabelle; Arnold, Susanne; Mujica-Mota, Ruben; Metcalfe, Andrew; Simpson, Hamish; Murray, David; McGonagle, Dennis; Sharma, Hemant; Hamilton, Thomas; Ellard, David; Fernandez, Catherine; Reynolds, Catherine; Harwood, Paul; Croft, Julie; Stocken, Deborah; Pandit, Hemant

This article was not externally reviewed at BMJ Open. Protocols that have been independently assessed prior to submission to BMJ Open are usually fast-tracked to publication on the grounds that further substantial changes will not be possible. This independent assessment will usually be external, independent review for both a substantial grant award from a non-commercial or government funder and ethics approval.
